# Banked Primary Progenitor Cells for Allogeneic Intervertebral Disc (IVD) Therapy: Preclinical Qualification and Functional Optimization within a Cell Spheroid Formulation Process

**DOI:** 10.3390/pharmaceutics16101274

**Published:** 2024-09-29

**Authors:** Annick Jeannerat, Cédric Peneveyre, Sandra Jaccoud, Virginie Philippe, Corinne Scaletta, Nathalie Hirt-Burri, Philippe Abdel-Sayed, Robin Martin, Lee Ann Applegate, Dominique P. Pioletti, Alexis Laurent

**Affiliations:** 1 Development Department, LAM Biotechnologies SA, CH-1066 Epalinges, Switzerland; annick.jeannerat@lambiotechnologies.com (A.J.); cedric.peneveyre@lambiotechnologies.com (C.P.); 2 Regenerative Therapy Unit, Plastic, Reconstructive and Hand Surgery Service, Lausanne University Hospital, University of Lausanne, CH-1066 Epalinges, Switzerland; sandra.jaccoud@chuv.ch (S.J.); virginie.philippe@chuv.ch (V.P.); corinne.scaletta@chuv.ch (C.S.); nathalie.burri@chuv.ch (N.H.-B.); philippe.abdel-sayed@chuv.ch (P.A.-S.); lee.laurent-applegate@chuv.ch (L.A.A.); 3 Laboratory of Biomechanical Orthopedics, Federal Polytechnic School of Lausanne, CH-1015 Lausanne, Switzerland; 4 Orthopedics and Traumatology Unit, Lausanne University Hospital, University of Lausanne, CH-1011 Lausanne, Switzerland; robin.martin@chuv.ch; 5 STI School of Engineering, Federal Polytechnic School of Lausanne, CH-1015 Lausanne, Switzerland; 6 Center for Applied Biotechnology and Molecular Medicine, University of Zurich, CH-8057 Zurich, Switzerland; 7 Oxford OSCAR Suzhou Center, Oxford University, Suzhou 215123, China

**Keywords:** allogeneic cytotherapies, back pain, chondrogenesis, cell therapy, hypoxia, intervertebral disc, manufacturing process, spheroids, spine, tissue engineering

## Abstract

**Background/Objectives:** Biological products are emerging as therapeutic management options for intervertebral disc (IVD) degenerative affections and lower back pain. Autologous and allogeneic cell therapy protocols have been clinically implemented for IVD repair. Therein, several manufacturing process design considerations were shown to significantly influence clinical outcomes. The primary objective of this study was to preclinically qualify (chondrogenic potential, safety, resistance to hypoxic and inflammatory stimuli) cryopreserved primary progenitor cells (clinical grade FE002-Disc cells) as a potential cell source in IVD repair/regeneration. The secondary objective of this study was to assess the cell source’s delivery potential as cell spheroids (optimization of culture conditions, potential storage solutions). **Methods/Results:** Safety (soft agar transformation, β-galactosidase, telomerase activity) and functionality-related assays (hypoxic and inflammatory challenge) confirmed that the investigated cellular active substance was highly sustainable in defined cell banking workflows, despite possessing a finite in vitro lifespan. Functionality-related assays confirmed that the retained manufacturing process yielded strong collagen II and glycosaminoglycan (GAG) synthesis in the spheroids in 3-week chondrogenic induction. Then, the impacts of various process parameters (induction medium composition, hypoxic incubation, terminal spheroid lyophilization) were studied to gain insights on their criticality. Finally, an optimal set of technical specifications (use of 10 nM dexamethasone for chondrogenic induction, 2% O_2_ incubation of spheroids) was set forth, based on specific fine tuning of finished product critical functional attributes. **Conclusions:** Generally, this study qualified the considered FE002-Disc progenitor cell source for further preclinical investigation based on safety, quality, and functionality datasets. The novelty and significance of this study resided in the establishment of defined processes for preparing fresh, off-the-freezer, or off-the-shelf IVD spheroids using a preclinically qualified allogeneic human cell source. Overall, this study underscored the importance of using robust product components and optimal manufacturing process variants for maximization of finished cell-based formulation quality attributes.

## 1. Introduction

Pathological and traumatic affections of the intervertebral disc (IVD) are often the cause of lower back pain (LBP) [1,2]. Importantly, LBP constitutes a widespread health issue, as it is the greatest cause of disability burden worldwide [3]. Specifically, it is expected that more than half of the global population will experience LBP at some point, wherein 5–10% of patients will develop chronic LBP [3,4,5]. As LBP prevalence increases with age, so do the incured economic impacts (i.e., treatment costs, reduced productivity, loss of income). Of note, the latter have been estimated at over 100 bn USD yearly, with ⅔ of indirect costs (e.g., professional productivity deficits) [6,7]. Notwithstanding, LBP has been identified as the most common cause of disability among young adults in the US and in Europe, wherein LBP prevalence increases are driven by population growth, obesity, and aging [5,8]. Pathophysiologically, IVD affections are implicated in >40% of cases of chronic back pain and constitute the most common non-cancer indication for opioid prescription in the US [9,10].

From an anatomical perspective, the IVD deploys a spacer function between vertebrae in the spine [9]. The inner core of IVDs (i.e., nucleus pulposus [NP]) is constituted by a gelatinous hydrophilic extracellular matrix (ECM), rich in ACAN and COL2 (i.e., a 20:1 ratio). Therein, NP cells are characterized by a chondroid phenotype and generate high ACAN contents, which swell and thus exert sufficient mechanical pressure to maintain appropriate distances between vertebral bodies. The NP is contained in the annulus fibrosus (AF) structure, which is a COL1-rich tissue presenting hyaline cartilaginous endplates [8,9,11]. As a unit, the IVD serves as a shock absorber within the spine, presenting resistance to tensile and torsional forces. As IVDs are mainly avascular and aneural, nutrients and metabolites diffuse from nearby vessels in the endplates and in the outer AF [2,9,12].

From a therapeutic standpoint, conservative LBP treatments primarily aim for pain relief, using physiotherapy, chiropraxie, acupuncture, NSAIDs, opiates, steroids, or muscle relaxants [13]. Surgical approaches (e.g., microdiscectomy, nucleoplasty, annuloplasty, spinal fusion, disc replacement) are indicated when patients do not respond to conservative treatment options. However, spine surgeries often increase the rates of adjacent segment degeneration [14]. Furthermore, patient self-rating of unsuccessful treatment after spinal fusion may exceed 30%, and complications were notably reported in 36% of cases [10,15].

Due to the complex pathophysiological context of IVD degeneration and the limited success of traditional treatment approaches, considerable interest and research have been focused on therapies wielding intradiscal medical device scaffolds and/or biologicals [14,16]. Therein, the objectives are to restore normal IVD functions, notably by enhancing ECM anabolic processes or by reducing the action of catabolic molecules (e.g., MMPs). Therefore, several growth factors (e.g., GDF-5), biologicals (e.g., platelet-rich plasma [PRP]), or cell-based therapeutics are clinically investigated [17,18,19,20,21,22]. While hyaluronan-based hydrogels and fibrin-based sealants are useful for AF repair or after nucleotomies, bioactive molecules (e.g., glucocorticoids, NSAIDs, anti-TNFs) are often used [23,24,25]. However, most IVD therapeutic interventions only allow for symptomatic management of pain and present limited efficacy in the long term.

Recently, considerable efforts have been allocated to investigational cell therapies and tissue engineering solutions aiming to regenerate IVDs [3,8,14]. Several autologous cell-based protocols have been developed for IVD treatment, comprising ASCs, B-MSCs, or disc chondrocytes as the cellular active substance [14,26,27,28,29,30]. In an allogeneic setting, juvenile chondrocytes, B-MSCs, discogenic cells (DiscGenics, Salt Lake City, UT, USA), umbilical stem cells, NP particulates (Vivex Biologics, Miami, FL, USA), or mesenchymal precursors (Mesoblast, Melbourne, Australia) have been proposed [3,14,28].

Importantly, in the specific case of cell-based therapies, patient selection methodology was described as highly important for appropriate treatment (i.e., moderate severity in disc degeneration), as mild or advanced lesions may not show measurable improvement [10,26,27,28]. From a regulatory viewpoint, in order to potentially circumvent some of the reported challenges in cell-based IVD therapies, the use of exosomes has recently been proposed [31]. Such approaches bare the potential to avoid the detrimental effects of harsh IVD environments on implanted cells, adverse tumorigenicity potential, unwanted cell differentiation, or the risk of host immune reaction, which classically limits the application of viable cells [31,32]. However, further research and long-term follow-up are required around such novel biological-derived cell-free protocols.

The primary aim of the present study was to qualify a clinical-grade cryopreserved primary progenitor cell source (FE002-Disc cells) as a potential cellular active substance in IVD therapy. The secondary aim of this study was to assess the formulation potential of FE002-Disc cells in chondrogenically induced spheroids. The primary hypothesis of this study was that the safety and functionality attributes of the considered primary cell source were adapted for the development of investigational cell therapies for IVDs. The secondary hypothesis of this study was that significant functional impacts on the finished products could be induced by modulation of the manufacturing process technical specifications. The significance of this study resided in the establishment of defined processes for preparing fresh, off-the-freezer, or off-the-shelf IVD spheroids using a preclinically qualified allogeneic cell source. Overall, this study confirmed the critical importance of extensively investigating both the cell source and the manufacturing processes in the context of investigational therapeutic product development (i.e., safety and quality enhancement).

## 2. Materials and Methods

### 2.1. Reagents and Consumables

The main reagents and consumables were as follows: DMEM culture medium, L-glutamine, TrypLE™, Opti-MEM™, dexamethasone, BCA assay kits, NuPAGE™ Bis-Tris 4–12% protein gels, β-mercaptoethanol, PMSF, microAmp fast 96-well reaction plates (Thermo Fisher Scientific, Waltham, MA, USA); X-gal powder (Chemie Brunschwig, Basel, Switzerland); papain (Sigma Aldrich, Buchs, Switzerland); FBS, VitCp, low melting point agarose (Merck, Darmstadt, Germany); human platelet lysate (HPL; Stemulate^®^, Sexton Biotechnologies, Indianapolis, IN, USA); penicillin-streptomycin (Biowest, Nuaillé, France); ITS II 100× (PAN-Biotech, Aidenbach, Germany); TGF-β3 (PeproTech, London, UK); Blyscan-sulfated glycosaminoglycan assay kits (BioColor, Carrickfergus, UK); telomerase activity quantification qPCR assay kits (ScienCell, Carlsbad, CA, USA); saccharose (PanReac AppliChem, Darmstadt, Germany); dextran 40,000 (Pharmacosmos, Wiesbaden, Germany); Lyoprotect bags (Teclen, Oberpframmern, Germany); lyophilization vials (Schott, Mainz, Germany); lyophilization stoppers (Datwyler, Altdorf, Switzerland).

### 2.2. Instruments and Equipment

Cell surface markers were analyzed on a BD Accuri™ C6 Plus FACS system (BD, Franklin Lakes, NJ, USA). Colorimetric and luminescence measurements were performed on a Varioskan LUX multimode plate reader (Thermo Fisher Scientific, Waltham, MA, USA). Telomerase activity assays were run on a QuantStudio 3 PCR Systems instrument (Thermo Fisher Scientific, Waltham, MA, USA). Immunohistochemistry imaging was performed on an inverted IX81 fluorescence microscope (Olympus, Tokyo, Japan). Gel imaging was performed on a Uvitec Mini HD9 gel imager (Cleaver Scientific, Rugby, UK). Sample lyophilization was performed in a LyoBeta Mini pilot freeze-dryer (Telstar, Terrassa, Spain).

### 2.3. Cell Sourcing and Cell Culture Media Composition

The FE002-Disc primary progenitor cell source used for this study consisted of banked primary human diploid cells from a clinical-grade source, as previously described [33]. The considered FE002-Disc primary progenitor cells were procured and produced under the Swiss progenitor cell transplantation program and were made available as cryopreserved stocks (TEC-PHARMA SA, Bercher, Switzerland). Briefly, a regulated organ donation at 14 weeks of gestation (i.e., FE002 donation) served for the establishment of the primary progenitor cell source (i.e., FE002-Disc cell type) used in the investigations presented herein. Full donor informed consent was obtained and confirmed for the organ donation and for the inclusion in the ad hoc progenitor cell transplantation program. In addition to extensive donor medical history screening, cytogenetic analyses, and histopathological investigations of the donated tissues, the donor was serologically tested twice (i.e., at the time of the donation and three months later) for specified pathogens (i.e., CMV, EBV, HBsAg, HBV, HCV, HIV-1, HIV-2, HSV, HTLV-1, HTLV-2, S-West Nile virus, *Toxoplasma gondii*, *Treponema pallidum*). Among other primary cell sources, primary progenitor IVD cells were isolated in vitro from the FE002 organ donation. The isolated IVD tissue biopsies were mechanically and/or enzymatically processed for the in vitro culture initiation of fibroblastic adherent primary progenitor cells (i.e., FE002-Disc cell types) in good manufacturing practice (GMP)-compliant manufacturing suites. Briefly, the procured IVD tissue samples were thoroughly washed in conserved phosphate-buffered saline (PBS) buffer (Bichsel, Interlaken, Switzerland), further dissected, and appropriately processed and conditioned for adherent primary progenitor cell proliferation initiation.

After the initial addition of adequate proportions of the cell culture medium (i.e., Dulbecco’s modified Eagle medium, DMEM, supplemented with 10% *v*/*v* fetal bovine serum, FBS, Gibco™ and Invitrogen™, respectively, Thermo Fisher Scientific [Waltham, MA, USA]), the cell culture vessels were incubated at 37 °C in humidified incubators under 5% *v*/*v* CO_2_. Following iterative cell culture medium exchange procedures, preliminary progenitor cell cultures were harvested by trypsinization (i.e., 0.05% trypsin-EDTA, Gibco™, Thermo Fisher Scientific, USA) and were further used to perform in vitro monolayer sub-cultures of FE002-Disc cells following the defined ad hoc technical specifications. Following appropriate maintenance and harvest of the primary cell sub-cultures, the obtained biological materials were cryopreserved in individual polymeric vials in a DMSO-containing cryopreservation solution for the establishment of FE002-Disc parental cell banks (PCB) at passage level 1. After appropriate testing, qualification, and quarantine release of the cryopreserved FE002-Disc PCB cellular material lots, these were used as the starting materials in defined serial expansion workflows in order to establish FE002-Disc master cell banks (MCB) and the FE002-Disc working cell banks (WCB).

For the present study, FE002-Disc cells from a WCB were initiated and were serially expanded in complete growth medium (CMtc) consisting of high-glucose DMEM supplemented with 2 mM L-glutamine and 10% *v*/*v* fetal bovine serum (FBS). The FE002-Disc cells were expanded in humidified incubators at 37 °C with 5% CO_2_ in normoxic (i.e., 21% O_2_) culture conditions or in hypoxic (i.e., 2% O_2_) culture conditions. The cultures were maintained until cell monolayers attained confluency, with cell culture medium exchange procedures performed twice weekly. Respective proliferation characteristics (i.e., cellular morphology, population doubling values) were determined between the normoxic and hypoxic culture conditions. The FE002-Disc cells were used between passage levels 5 and 14 in this study, depending on the assay setup. Primary epiphyseal chondroprogenitors (i.e., FE002-Cart.Art cell type) were used as controls, where the cells were maintained in the same way as the FE002-Disc cells and were used between passage levels 6 and 8 in this study [34].

Cryopreserved primary adipose-derived mesenchymal stem cells (i.e., ASC-F cell type, passage level 2, 10^6^ cells/vial) were purchased from ZenBio (Durham, NC, USA). The ASCs were isolated from human subcutaneous abdominal adipose tissue obtained from a competent 34-year-old female Caucasian donor (i.e., BMI 21.3 kg/m^2^, non-smoker, non-diabetic, non-medicated) undergoing elective surgery. Safety control results from the supplier indicated that the ASCs tested as negative for HIV, hepatitis B, and hepatitis C. Quality control results from the supplier indicated conforming results for cellular viability, trilineage differentiation potential (i.e., adipogenic, chondrogenic, osteogenic), and cell surface markers (i.e., 97.9% positive for CD105, 99.8% positive for CD44, 0.19% positive for CD19, and undetectable for CD31). The stem cells were initiated according to the supplier’s specifications and were seeded at 3.0 × 10^3^ cells/cm^2^ in T75 flasks. The stem cells were expanded in normoxic culture conditions in specific growth medium (CM-HPL) composed of high-glucose DMEM supplemented with 2 mM L-glutamine and 5% *v*/*v* human platelet lysate (HPL). The cultures were maintained until the cell monolayers attained confluency, with medium exchange procedures performed twice weekly. The ASCs were harvested and used for experiments at passage level 4.

The HeLa cell line was obtained from the Musculoskeletal Research Unit at the University of Zurich (Zurich, Switzerland). The cells were expanded in CMtc in normoxic conditions in a quarantine incubator. The cultures were maintained until the cell monolayers attained confluency, with cell culture medium exchange procedures performed twice weekly. The cells were harvested and used for experiments when they reached 100% confluency.

### 2.4. FE002-Disc Cellular Active Substance Preclinical Characterization Assays

The FE002-Disc cellular active substance was firstly analyzed in terms of composition, safety attributes, and biological function in order to preliminarily confirm its applicability for therapeutic product formulation. Specifically, the experiments focused on the proteomic composition of the cellular active substance, the in vitro behavior and robustness of the primary cells, and the in vitro exclusion of safety-related concerns (e.g., tumorigenicity or continuous cell line behavior).

#### 2.4.1. Proteomic Composition Screening in Multiplex Analyses

Soluble protein identification and quantification were performed on FE002-Disc cell lysates at Eve Technologies (Calgary, AB, Canada) using multiplex analyses. For sample preparation, 10^7^ cells were harvested from cultures, resuspended in 1 mL PBS, and were thermically lysed using the freeze-thaw method. The samples were then centrifuged at 13,000 rpm at 4 °C for 5 min, and the supernatants were collected. The obtained samples were analyzed by BCA for total protein quantification and were sent on dry ice for multiplex analyses. The retained multiplex kits were as follows: the human angiogenesis array and growth factor 17-plex array, the human cytokine/chemokine 96-plex panel, the human soluble cytokine receptor 14-plex array, the human MMP and TIMP panel for cell cultures, and the cytokine TGF-β 3-plex array. Based on the obtained proteomic data, specific protein concentrations in the samples were calculated by normalization to the total protein contents.

#### 2.4.2. Cell Surface Marker Characterization by Flow Cytometry

From an identity and purity standpoint, the cellular active substance was analyzed for cell surface marker expression. Therefore, cell surface marker characterization was performed by FACS analysis on FE002-Disc cells at passage level 6. The harvested cells were incubated with specific primary antibodies coupled to either FITC or PE fluorophores for 1 h. Selected cell surface marker antibodies were anti-human CD90, CD73, CD105, CD26, CD166, CD44, HLA-ABC, CD19, CD14, CD34, CD45, and HLA-DPQR. Antibody references are presented in Supplementary Methods. The samples were run on a BD Accuri™ C6 Plus FACS system, and data analysis was performed with the BD Accuri™ C6 software, v264.21.

#### 2.4.3. Phenotypic Stability Assessment in Chemical Induction Assays

From an identity and cell type stability standpoint, the cellular active substance was analyzed for potential phenotypic plasticity in chemical induction assays. Therefore, FE002-Disc cells were analyzed in terms of differentiation potential under adipogenic, osteogenic, and chondrogenic culture conditions. ASCs were included in the assays as positive differentiation controls in adipogenic and osteogenic induction. The effect of in vitro cellular passaging/aging on the differentiation potential of the FE002-Disc cells was evaluated by iteratively performing the experiments in triplicate at passage levels 6–11.

For adipogenic differentiation assays, the cells were seeded in 12-well plates and were maintained in culture in their respective media under normoxic conditions until 80% confluency was attained. The cells were then transferred and maintained in adipogenic induction medium, consisting of proliferation medium supplemented with ITS, 1 µM dexamethasone, 100 µM indomethacin, and 100 µM IBMX. After two weeks of induction, the cells were fixed in 4% formalin and were stained with Oil Red O for the revelation of lipid droplets.

For osteogenic differentiation assays, the cells were seeded in collagen 1-coated 12-well plates and were maintained in culture in their respective media under normoxic conditions until 50% confluency was attained. The cells were then transferred and maintained in osteogenic induction medium, consisting of DMEM with 5% HPL, supplemented with 80 µg/mL VitCp, 5 mM β-glycerophosphate, and 100 nM dexamethasone. After three weeks of induction, the cells were fixed in 4% formalin and stained with Alizarin Red.

For chondrogenic differentiation assays, 5 × 10^5^ cells were transferred in 15 mL tubes and were centrifuged for 5 min at 500× *g*. The formed cell pellets were then maintained in chondrogenic induction medium, consisting of DMEM supplemented with ITS II 1×, 100 nM dexamethasone, 10 ng/mL TGF-β3, and 82 µg/mL VitCp. The cell pellets were maintained in normoxic conditions for 4 weeks with medium exchanges performed three times per week before fixation in 4% formalin. Following sample inclusion and histology slide preparation, staining with Alcian Blue, anti-ACAN, and anti-collagen 2 antibodies was performed (Musculoskeletal Research Unit, University of Zurich, Zurich, Switzerland).

#### 2.4.4. β-Galactosidase Staining for In Vitro Cell Senescence Assessment

From a first safety evaluation standpoint, the β-galactosidase assay was retained in order to confirm that the FE002-Disc cells possess limited proliferation potential and become senescent over in vitro passaging (i.e., limited proliferation lifespan, excludes tumor cell behavior). Therefore, proliferating cells at passage levels 7 and 14 were fixed for 5 min in fixation solution (i.e., 1.85% formaldehyde with 0.2% glutaraldehyde) and were then rinsed with PBS 1×. Staining was performed at 37 °C with a SA-β-gal staining solution, consisting of 0.1% X-gal, 5 mM potassium ferrocyanide, 5 mM potassium ferricyanide, 150 mM NaCl, and 2 mM MgCl_2_ in a 40 mM citric acid/sodium phosphate solution at pH 6.0. Following overnight staining, the cells were washed, and the presence of β-galactosidase-positive (i.e., blue staining) cells was observed microscopically. Random field imaging (n = 8) was performed in contrast phase microscopy, and operator enumeration of senescent cells was performed.

#### 2.4.5. Telomerase Activity Quantification for In Vitro Tumorigenicity Assessment

From a second safety evaluation standpoint, an in vitro telomerase activity quantification assay was performed in order to confirm that the FE002-Disc cells present low levels of telomerase activity as compared to the tumorigenic HeLa cells. Therefore, a qPCR telomerase activity quantification kit was used to determine the relative level of telomerase activity in the samples. HeLa cells were used as positive controls, and FE002-Cart.Art cells were used as negative controls. The assay was performed according to the instructions of the manufacturer, wherein frozen dry cell pellets were used as starting materials. Cell lysis was performed by adding lysis buffer supplemented with PMSF and β-mercaptoethanol to the pellets, followed by a 30 min incubation on wet ice. The samples were centrifuged at 12,000× *g* at 4 °C for 20 min, and the supernatants were transferred to new Eppendorf tubes. For telomerase activity detection, the samples, telomerase reaction buffer, and nuclease-free water were mixed and incubated at 37 °C for 3 h. The reaction was quenched by heating the samples at 85 °C for 10 min. The qPCR reactions were prepared by mixing the quenched samples, primers, TaqGreen qPCR master mix, and nuclease-free water. The qPCR run conditions comprised an initial denaturation step of 10 min at 95 °C and 36 amplification cycles (i.e., denaturation over 20 s at 95 °C; annealing over 20 s at 52 °C; extension over 45 s at 72 °C). The samples were run in triplicate. Resulting Ct values > 33 were assessed as being negative. Relative telomerase activity quantification between the HeLa cells and the included primary cells was based on the 2^–ΔΔCt^ calculation method.

#### 2.4.6. Soft Agarose Colony Formation Assay for In Vitro Semi-Quantitative Tumorigenicity Assessment

From a third safety evaluation standpoint, a standard soft agarose cell colony formation assay was used to assess the potential of the FE002-Disc cells to proliferate in non-adherent settings (i.e., a characteristic of tumoral cells). The assays were performed in triplicate in 24-well microplates. The solid agarose layer (i.e., bottom layer) was composed of 0.6% agarose in PBS- and FBS-supplemented growth medium with 1% penicillin-streptomycin. The soft agarose layer (i.e., top layer) was composed of 0.4% agarose and contained the investigated cellular materials (i.e., 500–10^4^ viable cells/well). The cells had been freshly harvested from confluent cultures. CMtc with 1% penicillin-streptomycin was added on top of the soft agarose layer, and the assay plates were incubated at 37 °C under 5% CO_2_ (i.e., normoxic and hypoxic conditions) in a humidified incubator for 21 days. The plates were regularly microscopically assessed, and representative imaging was performed (i.e., days 1, 4, 7, 21) to comparatively assess the formation of non-adherent cell colonies.

#### 2.4.7. Timecourse of HIF-1 Induction with Western Blotting Readout

The hypoxic incubation condition retained for this study (i.e., 2% O_2_ level) was designed to mimick the local hypoxic environment of the IVD structure. In order to assess the impact of incubation condition modification (i.e., from normoxia during the cell amplification phase to hypoxia for the subsequent spheroid manufacturing phase), a HIF-1 induction assay was performed on cell monolayers. Therefore, FE002-Disc cells at passage level 6 were thawed, seeded in 6-cm culture dishes at 3 × 10^3^ cells/cm^2^ in CMtc, and maintained in normoxic conditions. When the cells reached 90% confluency, the plates were transferred in hypoxic culture conditions. The cells were then lysed in RIPA lysis buffer supplemented with proteinase inhibitors at different time points and were stored at –20 °C until analysis. Total protein levels in the samples were quantified using a BCA assay. Then, 10 µg of total protein/sample were separated by electrophoresis on 4–12% Bis-tris polyacrylamide gels and were transferred onto a nitrocellulose membrane. The membrane was incubated overnight at 4 °C with primary anti-HIF-1α (BD Biosciences, Franklin Lakes, NJ, USA) and anti-actin (Thermo Fisher Scientific) antibodies. The following day, the membrane was incubated with the corresponding secondary antibody (i.e., anti-mouse-HRP or anti-rabbit-HRP). Revelation was performed with the ECL™ Prime chemiluminescence detection system.

#### 2.4.8. Inflammatory Challenge Assays

In order to assess the behavior and resilience of the FE002-Disc cells in inflammatory environments (e.g., degenerating IVDs), an in vitro inflammatory challenge assay was performed. Therefore, FE002-Disc cells at passage level 5 were seeded at 5 × 10^3^ cells/cm^2^ in 24-well plates. The cells were maintained in CMtc in normoxic or hypoxic conditions. After 8 days in culture, the medium was replaced by CMtc supplemented with TNF-α at doses ranging from 0 to 100 ng/mL. The assay plates were incubated again for 24 h. Then, cellular morphology was recorded, and the levels of IL-6 and IL-8 secretion in the assay medium were determined by ELISA (Peprotech, Cranbury, NJ, USA).

### 2.5. FE002-Disc Cell Spheroid Manufacture Optimization and Functional Controls

Therapeutic cell-based products (i.e., cell suspensions or cell spheroids) have been studied for intra-NP delivery in IVD affections. Using the FE002-Disc cellular active substance, finished product prototypes in the form of chondrogenically induced cell spheroids were manufactured, controlled, and optimized for quality and function (e.g., aggrecan deposition and collagen II expression), as described in the following sub-sections.

#### 2.5.1. Cell Spheroid Manufacture under Hypoxia

From a function-oriented manufacturing optimization standpoint, the effects of the chondrogenic induction medium composition and incubation atmosphere on cell spheroid formation were assessed. Therefore, FE002-Disc cells were seeded in cell-repellent 96-well plates using 20–40 × 10^3^ cells per well and spontaneously formed cell spheroids overnight. The spheroids were maintained in CMtc or in chondrogenic differentiation medium variants (i.e., dexamethasone concentrations of 1–100 nM) and in normoxic or hypoxic conditions. The plates were maintained in culture for three weeks with medium exchanges performed three times per week. Microscopic imaging was performed once a week (i.e., documenting spheroid size evolution over time).

#### 2.5.2. Cell Spheroid Cryopreservation and Lyophilization

In order to potentially obtain off-the-freezer or off-the-shelf spheroid formulations, pilot cryopreservation and lyophilization protocols were investigated. Therefore, large FE002-Disc spheroid batches were prepared with a chondrogenic induction period of three weeks. For the cryopreservation arm, groups of 4 spheroids were suspended in cryotubes in a freezing solution composed of 50% CMtc, 40% FBS, and 10% DMSO. Controlled-rate freezing was performed in a CoolCELL device placed at –80 °C overnight, followed by transfer to liquid nitrogen storage. For the lyophilization arm, groups of 4 spheroids were suspended in 2R clear glass vials in a lyopreservation solution composed of 8% *m*/*v* saccharose and 2% *m*/*v* dextran 40,000 in a buffered injection-grade solvent. Freezing was performed overnight at –20 °C. Primary drying was performed overnight at a product temperature of 7 °C under 0.08 mbar. Secondary drying was then performed for 6 h at a product temperature of 30 °C under 0.01 mbar. The lyophilized samples were sealed and stored at 4 °C until use.

#### 2.5.3. DMMB Quantification for Assessment of ECM Deposition

From a first chondrogenic function assessment viewpoint, total GAG deposition in the spheroids was quantified using a DMMB kit according to the manufacturer’s protocol. Briefly, 4 spheroids were harvested for each condition, washed once with PBS, and were incubated for 3 h in papain digestion buffer until complete lysis. The samples were centrifuged at 10,000 rpm for 10 min, and the supernatants were transferred into new Eppendorf tubes. Total GAG precipitation was obtained by mixing the samples or standard with the dye reagent, followed by 30 min of incubation at ambient temperature. The samples were then centrifuged at 12,000 rpm for 10 min and were carefully inverted to discard all the supernatant. A dissociation reagent was added to the GAG pellets to release the dye, and the samples were vortexed until complete dye dissociation. The samples were centrifuged at 12,000 rpm for 5 min. Finally, 100 µL of standard or sample were transferred to 96-well plates, and the absorbance value was measured at a wavelength of 656 nm. Total GAG contents in the samples were determined using a dye standard curve.

#### 2.5.4. Western Blotting and Immunohistology for Specific ECM Component Visualization

From a second chondrogenic function assessment viewpoint, cartilage oligomeric matrix protein (COMP) expression evaluation in the spheroids was performed by Western blotting. Therefore, 4 spheroids for each condition were pooled, treated with RIPA lysis buffer supplemented with protease inhibitors, and sonicated. The samples were then incubated for 15 min on wet ice, centrifuged at 13,000 rpm for 5 min, and the supernatants were transferred into new Eppendorf tubes. The samples were then separated by electrophoresis on 4–12% Bis-tris polyacrylamide gels and were transferred onto nitrocellulose membranes. The membranes were incubated overnight at 4 °C with the primary anti-COMP (Abcam, Cambridge, UK) and anti-actin (Abcam) antibodies. The following day, the membranes were incubated with the anti-rabbit-HRP secondary antibody. Revelation was performed with the ECL™ Prime chemiluminescence detection system.

From a third chondrogenic function assessment viewpoint, ECM components of the spheroids were evidenced using direct staining and immunohistology. Therefore, at the end of the manufacturing phase, the spheroids were harvested and fixed overnight at 4 °C in a 4% formalin solution. The samples were then rinsed three times with PBS and were transferred to 70% EtOH at 4 °C until inclusion in paraffin. Thin 5-μm sections were cut and stained for ECM components (e.g., ACAN, COL2).

### 2.6. Statistical Analysis and Data Presentation

For the statistical comparison of average values from two sets of data, a paired Student’s *t*-test was applied, following an appropriate evaluation of the normal distribution of the data. A *p*-value < 0.05 was specified for statistical significance determination. The calculations and data presentation were performed using Microsoft Excel v16.0, Microsoft PowerPoint v16.0 (Microsoft Corporation, Redmond, WA, USA), and GraphPad Prism version 8.0.2 (GraphPad Software, San Diego, CA, USA). Physical descriptions of the spheroid parameters (e.g., circularity, roundness, Feret’s diameter) were calculated using the ImageJ software v1.54k (National Institutes of Health, Bethesda, MD, USA).

## 3. Results

### 3.1. Identity and Compositional Attributes of the FE002-Disc Progenitor Cell Source

As the FE002-Disc cell source was not previously extensively characterized, the first area of experimental interest from an identity standpoint consisted in cell surface markers. Therein, FE002-Disc cells presented a panel of cell surface markers similar to that of the previously published FE002-Cart.Art cell source, but also to the FACS profiles described for NPSCs [35,36]. Specifically, FE002-Disc cells were found to be positive for CD73, CD90, CD105, CD26, CD44, CD166, and HLA-ABC markers (Table S1). Furthermore, they were negative for the endothelial cell marker CD34 and for the immune cell markers CD19, CD45, CD14, and HLA-DPQR (Table S1). Of note, no difference in cell surface marker expression was evidenced between cell culture conditions (i.e., normoxic versus hypoxic cell expansions, Table S1). Globally, the obtained FACS data confirmed that the FE002-Disc cells were close to alternative progenitor or stem cell sources in terms of surface marker expression while presenting an appropriate immunological profile for therapeutic allogeneic applications (Table S1) [35,36].

From an active substance composition and function standpoint, the FE002-Disc cell source was investigated using proteomic analyses. Importantly, one of the predominantly described mechanisms of action (MoA) for cell-based therapies is the paracrine effect of the transplanted cells [37,38,39]. Thus, multiplex analyses were performed on cell lysate soluble fractions in order to identify potentially active proteins/factors to be used as biochemical markers. The samples were analyzed using kits targeting MMPs/TIMPS, angiogenesis and growth factors, TGF-β, cytokines, chemokines, and soluble cytokine receptors. The most abundant proteins belonged to the MMP/TIMPs, growth factors, and soluble receptor families (Table S2). Furthermore, the investigated soluble fractions contained only low amounts of cytokines and chemokines (Table S2). Interestingly, no log_10_ difference between normoxia and hypoxia culture conditions was evidenced for these proteinic targets (Table S2).

From a more detailed mechanistic breakdown viewpoint, several identified components of the cellular active substance were found to be relevant in an IVD treatment context, as detailed hereafter (Table S2). Notably, increased MMP levels were evidenced in cases of degenerated IVD [12]. Conversely, TIMPs are known to be expressed at low levels in non-degenerated IVD tissue [12]. Thus, the presence of TIMPs in therapeutic biological formulations may participate in the regulation of endogenous MMPs for reduction of ECM degradation. Similarly, the in vivo therapeutic use of HGF was reported to alter matrix catabolism but without promoting anabolism [16]. Of note, IL-6 is upregulated in degenerated IVDs; therefore, downregulation of IL-6 signaling by sgp130 could potentially reduce the degenerative process (Table S2). Furthermore, sTNFR (i.e., natural TNF scavenger) may help to regulate the negative effects (e.g., induced production of MMPs and ILs, cellular apoptosis) of overexpressed TNF in IVD affections [40]. Overall, while proteomic composition is a highly limited functional proxy for cell therapies and tissue engineering products, the presence of factors and proteins with confirmed roles in IVD pathophysiology was interpreted positively from a quality standpoint (e.g., specific active substance functional markers; Table S2).

### 3.2. In Vitro Lifespan of FE002-Disc Progenitor Cells: Quality-Guided Selection of Appropriate Passage Levels

A major advantage of using an allogeneic therapeutic cell source resides in the possibility of preparing standardized products without the need for patient-specific starting material harvest biopsies. The use of primary cells (e.g., FE002-Disc cell source) inherently implies that a defined in vitro cellular lifespan must be characterized and considered for manufacturing (i.e., cell banking, product formulation) technical specification establishment. In detail, primary cells should not be used as an active substance at advanced in vitro passage levels (i.e., end of production passage levels), where loss of quality and functionality attributes may be expected due to the onset of cellular senescence. Thus, a large portion of manufacturing process development was based on iterative quality and functionality assays, performed throughout the in vitro cellular lifespan of the FE002-Disc cells while using culture condition variants.

**Figure 1:**
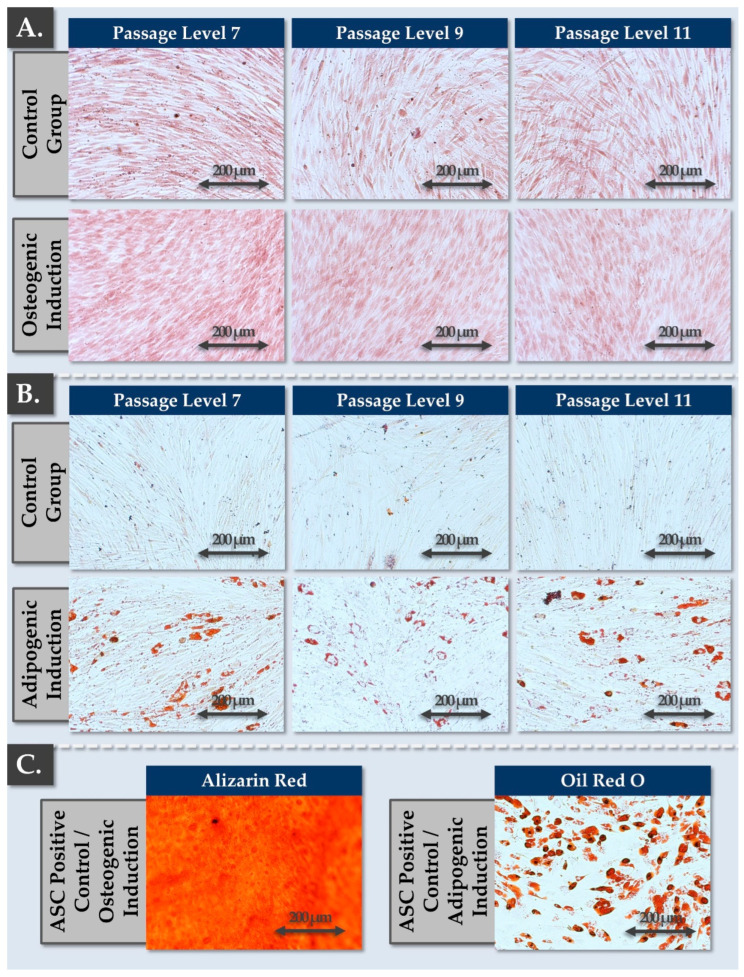
Results of iterative FE002-Disc progenitor cell phenotype plasticity assessments in chemical osteogenic and adipogenic induction studies. (**A**) No osteogenic differentiation was observed following Alizarin Red staining. (**B**) Mild adipogenic differentiation (i.e., lipid droplet accumulation upon differentiation in some of the cells in the population but not in all cells) was observed following Oil Red O staining. (**C**) Illustration of positive control groups for osteogenic (Alizarin Red staining, saturated with colorant due to strong cell differentiation) and adipogenic (Oil Red O staining) differentiation, respectively. Positive controls were prepared using ASC cultures, which were submitted to the chemical induction protocols. Scale bars = 200 µm. ASC, adipose stem cells.

The critical parameter linking in vitro cellular lifespan and functional stability attributes of the FE002-Disc cell source was phenotypic stability, or the sensitivity of the cells towards chemical induction. Firstly, two-dimentional osteogenic and adipogenic differentiation assays confirmed that FE002-Disc cells behaved consistently throughout serial subculturing (i.e., passage levels 7–11, Figure 1).

In detail, the FE002-Disc cells were shown to produce no mineralized matrix under osteogenic induction, while strong AR staining was observed in ASC controls after 3 weeks of differentiation (Figure 1A,C). Parallelly, mild lipid droplet accumulation within the primary progenitor cells was observed in adipogenic induction, but to a much lower extent than in the ASC controls (Figure 1B,C). This specific behavior (i.e., consistent throughout passages) was potentially linked to the presence of a small and persistent fraction of adipogenic progenitor cells (i.e., low amount, not capable of osteogenesis in this system) in the considered progenitor cell population. Alternatively and more probably, this behavior may be explained by the known properties of NP cells, which intrinsically present some phenotypic plasticity [41]. Importantly, the absence of phenotypic shifts throughout passages in monolayer adipogenic and osteogenic induction conditions confirmed the stability of the cell population toward adherent state differentiation (Figure 1).

**Figure 2:**
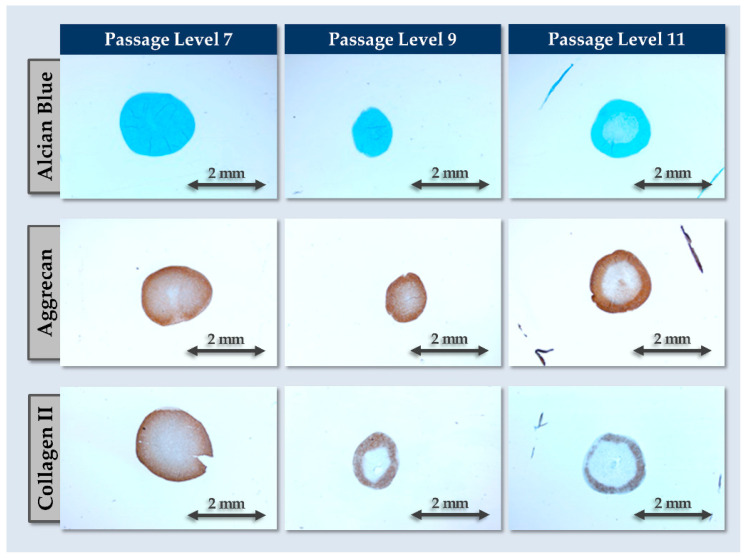
Results of iterative FE002-Disc progenitor cell phenotype plasticity assessment in chemical chondrogenic induction studies. The assays were carried out on large cell pellets in order to assess the intrinsic decline in chondrogenic potential over passage levels. Positive chondrogenic differentiation was observed throughout passages following staining of standard markers of cartilage ECM components (i.e., Alcian Blue staining for GAGs or immunohistochemical staining for aggrecan and collagen II). Scale bars = 2 mm. ECM, extracellular matrix; GAG, glycosaminoglycan.

Secondly, the chondrogenic differentiation potential of the FE002-Disc cells and the stability thereof were investigated in a three-dimensional (i.e., cell pellets) chemical induction setup. Therein, the chondrogenic potential of the cellular active substance was confirmed (Figure 2).

Specifically, cell pellets maintained in 3D in chondrogenic induction medium presented GAG accumulation (i.e., AB positive staining) and were positive for ACAN and COL2 deposition, which are characteristic NP proteins (Figure 2). Thus, the cells were found to regain chondrogenic differentiation potential and activity in 3D (e.g., induction and deposition of COL2), which is transiently lost during 2D cell expansion. However, a strong impact of in vitro cell aging (i.e., through serial passaging) was observed on the chondrogenic differentiation potential of the cells. In detail, a reduction in pellet size, a reduction in COL2 and ACAN expression, and a reduction in pellet staining homogeneity were recorded after passage level 7 (Figure 2). Thus, while FE002-Disc cells were shown to proliferate homogeneously in vitro in monolayers at least until passage level 11, the investigated functional attributes technically limited their further use to passage levels ≤ 7 (Figure 1 and Figure 2). Finally, it is noted that the retained readouts and specific stains used in the cellular active substance qualification assays corresponded to the current state-of-the-art of characterization and validation of chondrogenic cells for cell and gene therapies (Figure 2) [42].

### 3.3. FE002-Disc Progenitor Cell Resilience in Hypoxic and Inflammatory Environments

The degenerative IVD environment is known to be particularly harsh, with low oxygen and nutrient supplies, inflamed and acidic microenvironments, and important biomechanical constraints [9,24,25]. Therefore, several in vitro experiments were performed in order to assess the resilience of FE002-Disc cells placed in artificially challenging conditions. Such assays aimed to preliminarily determine if the considered progenitor cells were capable of withstanding hypoxic or inflamed environments.

The choice was made to work in a hypoxic environment in the present study in order to approximate the native physiological environment of the IVD cells. Specifically, the human IVD is avascular, resulting in an O_2_ gradient from the disc exterior region to the disc interior region (i.e., O_2_ levels reaching down to 1%) [43]. Furthermore, as in vitro hypoxic studies are often conducted between 1–5% O_2_, the value of 2% O_2_ was selected herein as a standardized condition in order to mimic a relatively strong hypoxia level that is coherent with physiological tissues [43]. The anticipated effect of hypoxia as regards the behavior of the FE002-Disc cells was initially a reduction of cellular proliferation or function, based on the reduced availability of oxygen, similar to native IVD tissue.

**Figure 3:**
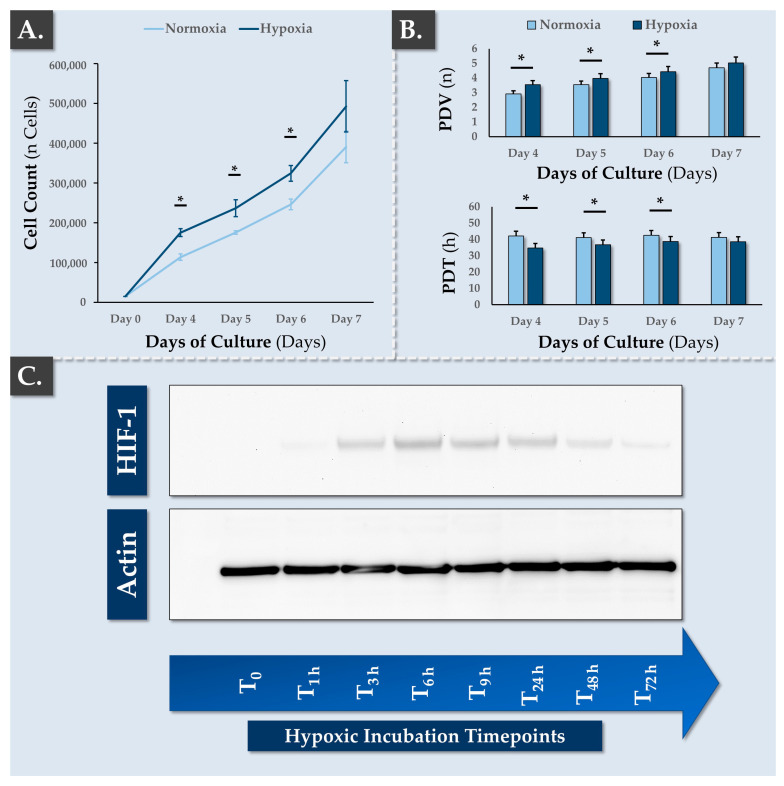
Results of FE002-Disc progenitor cell biological responses to hypoxic incubation conditions (i.e., 2% O_2_). (**A**) Comparative growth curves revealed significantly enhanced cellular proliferation in hypoxic conditions compared to normoxic conditions. (**B**) The significant increase (i.e., *p*-value < 0.05, identified by an asterisk “*”) in cellular proliferation under hypoxia was confirmed by analysis of the population doubling values and population doubling times. (**C**) Results of a timecourse HIF-1 detection assay with Western blot revelation. Transient HIF-1 induction was recorded during the first days of primary progenitor cell incubation in a hypoxic environment. Whole-gel imaging is presented in Figure S2. HIF, hypoxia-inducible factor; PDT, population doubling time; PDV, population doubling value.

Firstly, the effects of hypoxic incubation conditions on FE002-Disc cell survival and cell proliferation were quantified. Therefore, good monolayer cell adhesion and cellular proliferative morphology were observed in hypoxic culture conditions as compared to normoxic controls (Figure S1). No apparent cell death (i.e., abnormal morphology, cell detachment) or distress was recorded, and cell proliferation was statistically significantly higher in hypoxic environments as compared to normoxia until day 6 of culture (Figure 3A and Figure S1).

Of note, no statistically significant differences were found between the groups at the final timepoint of the assay (i.e., day 7 of culture), which was linked to the presence of confluent cells in the hypoxic group (Figure 3A,B). This factor probably inhibited the cellular proliferation for hypoxic cultures, while normoxic cultures were still sub-confluent at the 7-day timepoint. Namely, once the cells reached confluency, their proliferation probably slowed, this event occurring earlier for the hypoxic condition. From a biochemical standpoint, the FE002-Disc cells, which were maintained in normoxia, did not express HIF-1, as expected (Figure 3C). However, cell exposure to hypoxia led to a rapid response by stabilizing the HIF-1α protein and inducing signaling cascades. Consequently, HIF-1 expression was detected as early as 1 h following cell culture transfer from normoxia to hypoxia (Figure 3C). Globally, HIF-1 induction was recorded to be transient, as a progressive reduction in HIF-1 expression was observed after 48 h and 72 h of culture in hypoxia (Figure 3C).

**Figure 4:**
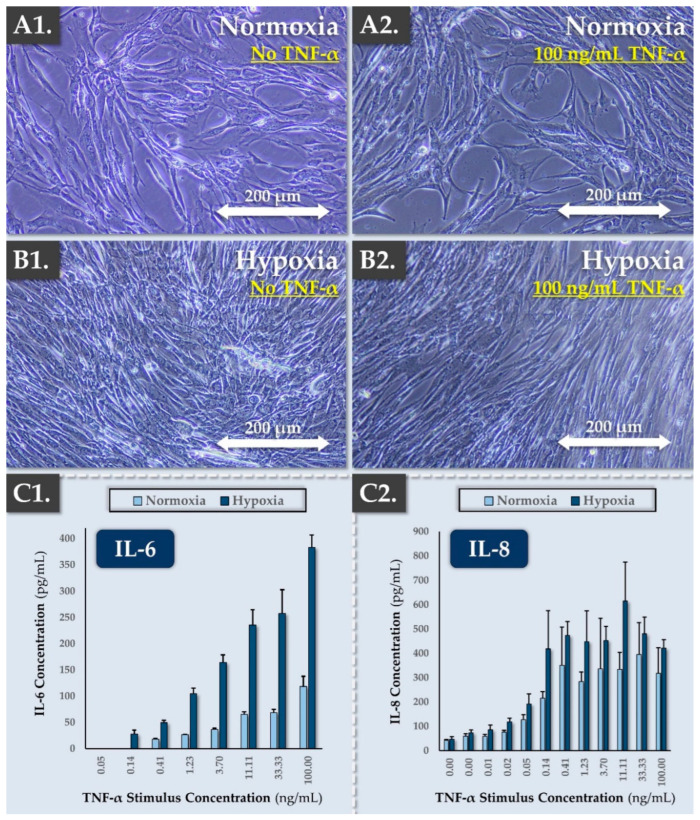
Results of FE002-Disc progenitor cell biological responses to a chemical inflammatory stimulus in normoxic and hypoxic culture conditions. (**A1**,**A2**) Normal cellular morphology (i.e., absence of toxicity signs) following expansion in normoxia, with and without TNF-α stimulation. Scale bars = 200 µm. (**B1**,**B2**) Normal cellular morphology (i.e., absence of toxicity signs) following expansion in hypoxia, with and without TNF-α stimulation. Scale bars = 200 µm. (**C1**,**C2**) ELISA-based quantification of total IL-6 and IL-8 following TNF-α stimulation of cells expanded in normoxia and in hypoxia, respectively. IL, interleukin; TNF, tumor necrosis factor.

As previously mentioned, inflammation is a hallmark of the degenerative IVD environment [44,45]. Therefore, therapeutic cells of cell-based constructs are mandatorily exposed to such adverse stimuli following transplantation. The resilience and behavior of the FE002-Disc cells were thus experimentally studied in an in vitro model of inflammation. Progenitor FE002-Disc cells were treated with increasing doses of TNF-α (i.e., up to 100 ng/mL) for 24 h and were maintained in normoxic or hypoxic conditions. Cellular morphology was recorded, and the secretion of IL-6 and IL-8 by the cells was quantified by ELISA (Figure 4).

In detail, the goal of the assay was to determine the cells’ behavior upon exposure to TNF, which was retained as a proxy for the hostile environment of the degenerated IVD. Specifically, TNF (i.e., multifunctional pro-inflammatory factor) is an activator of IVD degeneration (e.g., inflammatory response, cellular apoptosis). Indeed, TNF levels are increased in the nucleus pulposus of patients with degenerating IVDs, and it promotes the progression of the disease [46]. Notably, TNF injection in a porcine intervertebral disc model led to degenerative changes in the disc (i.e., decreased disc height, formation of fissures) [46].

Importantly, no changes in cellular morphology and no cellular detachment were observed after 24 h of TNF-α treatment in both incubation conditions, as observed in microscopy images of the cells (Figure 4A,B). In the absence of TNF-α treatment, no IL-6 secretion was observed, while basal IL-8 levels were already detected (Figure 4(C1,C2)). Furthermore, TNF-α stimulation was shown to induce IL-6 and IL-8 secretion in a dose-dependent manner (Figure 4C). In the TNF-α-stimulated groups, no shift in the IL induction curves was observed between normoxic and hypoxic culture conditions (Figure 4C). Of note, the absolute values of IL-6 and IL-8 in the hypoxic condition may have partly resulted from the increased cellular proliferation induced by hypoxia or from cellular potentiation by hypoxia (Figure 4C). Specifically, as the absolute values of secreted IL probably resulted from a complex effect (i.e., presence of more cells in the hypoxic group, presence of metabolically different cells in the hypoxic group), the normalization of IL levels to the cell counts was not performed. Thus, the observed increase in IL-6 and IL-8 levels could not be individually and directly attributed to the use of hypoxic incubation conditions versus the use of normoxic incubation conditions in this experimental setup (Figure 4C). Notwithstanding, TNF stimulation resulted in statistically significant (i.e., *p*-value < 0.05) increases in IL-6 and IL-8 levels in the assay cultures at all tested concentration ranges (Figure 4C).

Finally, even though EC_50_ values could not be experimentally calculated for IL-6 (i.e., the upper plateau was not defined), it was apparent that the IL-8 EC_50_ value was inferior to that of IL-6 (Figure 4C). Globally, the obtained datasets confirmed that the FE002-Disc cells were resilient in models of hypoxic environment and inflammation (Figure 3 and Figure 4). Such attributes were interpreted positively from a physiological function viewpoint for IVD progenitor cells and from a therapeutic standpoint in view of cell implantation in adverse in vivo microenvironments.

### 3.4. FE002-Disc Progenitor Cell Source In Vitro Safety Characterization

The therapeutic use of viable exogeneous cells or cell-based products inherently comports a risk of adverse effects, among which the formation of tumors. Therefore, thorough preclinical safety evaluation of specific cell sources is a prerequisite for any therapeutic product development process, especially for substantially manipulated materials (e.g., culture-expanded cells). For preliminary safety evaluation of the FE002-Disc cell source and following the principle of 3R rules, multiparametric in vitro analyses were performed [47].

Firstly, the FE002-Disc cell source was confirmed to possess a finite in vitro lifespan by experimental verification of cellular senescence attainment over serial passaging. A significant increase in cell PDT was observed between passage levels 6 and 13 (i.e., 92.4 h versus 66.7 h), along with a corresponding decrease in PDV (i.e., 3.60 versus 4.97).

Secondly, β-galactosidase staining demonstrated a statistically significant increase in the presence of senescent cells (i.e., positive blue cells) at high passage levels as compared to lower cell passage levels (i.e., 12.1% positive cells at passage level 6, 44.9% positive cells at passage level 13). Of note, no significant differences were outlined in terms of cellular senescence between the normoxic and the hypoxic culture conditions (Figure S3).

Thirdly, telomerase activity was quantified in FE002-Disc cells and was compared to that of the cancerous HeLa cell line (i.e., positive control) and that of FE002-Cart.Art cells (i.e., negative control). Experimentally, telomerase activity was detected in both of the included progenitor cell types but to a much lower extent than in HeLa cells (Figure S4). In detail, FE002-Disc cells presented telomerase activity levels higher than those of FE002-Cart.Art cells but similar to those of FE002-Ten primary progenitor tenocytes [34,48]. As no in vivo adverse effects (i.e., specifically with regard to tumor formation) were reported for both the FE002-Cart.Art and the FE002-Ten cells, the obtained telomerase activity data for the FE002-Disc cells was considered to meet expectations from a safety viewpoint [34,48].

Finally, soft agarose colony formation assays were performed in order to experimentally exclude the presence of tumorigenic behavior of the FE002-Disc cells in non-adherent culture conditions. Therein, positive control HeLa cells showed rapid colony formation and colony growth already at day 7 after cell seeding (Figure S5C). Namely, the HeLa cultures were stopped at day 14, as the cell colonies were becoming too large for the retained model. Conversely, no colony formation was observed in the FE002-Disc progenitor cell groups (i.e., normoxia and hypoxia conditions), even though ten times more cells were seeded per well (Figure S5A,B).

Overall, the presented safety-related experiments confirmed that the FE002-Disc cells reached senescence over serial in vitro passaging, presented low levels of telomerase activity, and were incapable of anchorage-independent cellular proliferation. While further in vivo tumorigenicity experiments are warranted to confirm the preclinical safety of the FE002-Disc cell source, the compiled in vitro data reported herein does not exclude such biological materials from further translational research based on safety-related attributes.

### 3.5. FE002-Disc Cell Spheroid Formulation Process and Function-Guided Optimization

Based notably on the use of spheroids/microtissues for the treatment of knee cartilage defects, several similar formulations were studied for IVD applications [49,50,51,52,53,54]. This approach is notably set forth to protect the transplanted cells in the adverse target implantation environment, as well as to directly supplement the administration site with exogeneous ECM (e.g., ACAN, COL2) [52,55,56]. Therefore, based on the functional and safety data gathered on the FE002-Disc cells in monolayer culture (i.e., cellular active substance), three-dimensional finished product prototypes in the form of cell spheroids were prepared and studied. Experiments were based on the reported effects of medium composition and incubation conditions on the chondrogenic potential of expanded articular chondrocytes [57,58,59,60,61,62].

Preliminary experiments demonstrated that the FE002-Disc progenitor cells could spontaneously form cell spheroids in low-attachment plates (Figure S6). Therein, the cell spheroids were maintained in culture for 14 days in normoxic or hypoxic environments and in the presence of complete growth medium or chondrogenic induction medium. The most significant increase in spheroid size over time was observed during the second week of culture, when chondrogenic medium and hypoxia culture conditions were combined (Figure S6B). Thus, for all further assays, the use of chondrogenic medium and hypoxic incubation conditions was retained.

**Figure 5:**
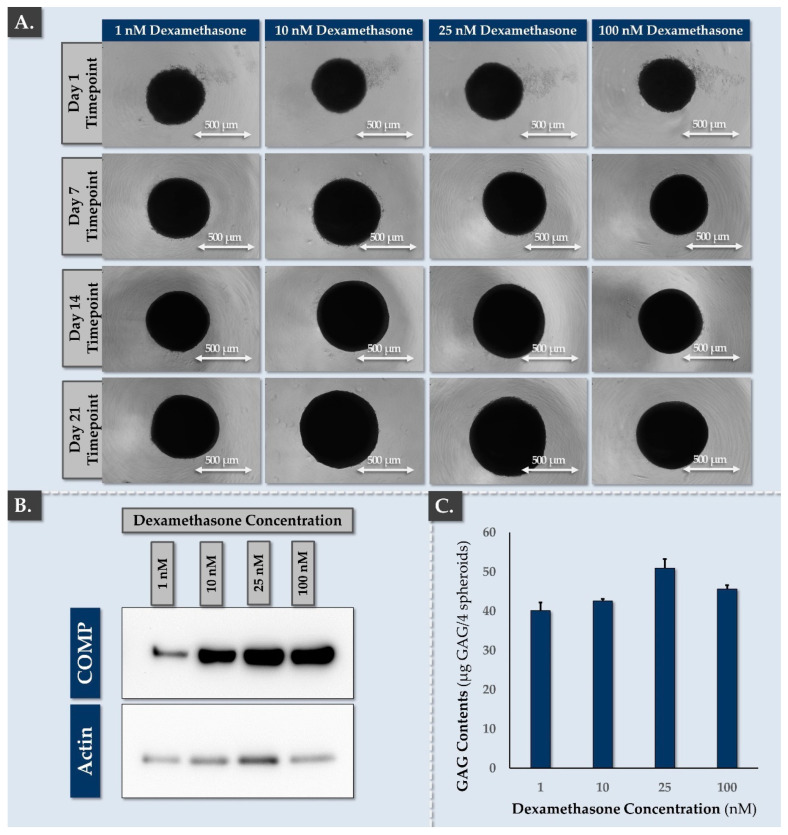
Results of FE002-Disc progenitor cell spheroid functional optimization assays, with comparative multiparametric assessment of various dexamethasone concentrations in the chondrogenic induction medium. (**A**) Comparative macroscopic assessment of the effect of dexamethasone concentration on cell spheroid size and morphology at various timepoints. Scale bars = 500 µm. (**B**) Comparative endpoint assessment of the effect of various dexamethasone concentrations on the endpoint expression level of COMP, with Western blot revelation. Whole-gel imaging is presented in Figure S7. (**C**) Comparative endpoint assessment of the effect of various dexamethasone concentrations on the quantity of total GAGs in the cell spheroids. COMP, cartilage oligomeric matrix protein; GAG, glycosaminoglycans.

Once the basic spheroid formulation process was established, further function-based optimization campaigns were carried out. Firstly, the dexamethasone concentration in the chondrogenic induction medium was optimized. Therein, a macroscopic increase in spheroid size was recorded across all groups between days 6–20, but the 1 nM and 100 nM dexamethasone concentrations tended to yield smaller spheroids (Figure 5A).

From a specific quantitative standpoint, cartilage oligomeric matrix protein (COMP) endpoint expression in the spheroids was strongly reduced when 1 nM dexamethasone was used, but it was otherwise consistent (Figure 5B). Of note, COMP is an ECM glycoprotein originally identified in cartilage and which plays a notable role in chondrogenesis [63]. For a broader quantitative overview of the deposited ECM in the studied spheroids, total GAG quantification was performed. The results showed that GAG contents were influenced by the dexamethasone concentration, where the highest GAG content was observed with 25 nM dexamethasone (Figure 5C). Specifically, the statistical analysis revealed that the GAG contents were significantly higher (i.e., *p*-value < 0.05) at 25 nM compared to 100 nM and significantly lower (i.e., *p*-value < 0.05) at 1 and 10 nM compared to 100 mM (Figure 5C). However, reducing the dexamethasone to concentrations as low as 1 nM still resulted in relatively high GAG contents, suggesting that the standard dexamethasone concentration (i.e., 100 nM) could be reduced in the spheroid production process (e.g., 1–25 nM).

From a physical characterization viewpoint, analysis of the spheroids produced with 10 and 100 nM dexamethasone (i.e., optimized condition and reference condition, respectively) indicated that the area of the spheroids and their Feret’s diameter increased over time in culture and that appropriate circularity and roundness values were consistently obtained (Table 1).

**Figure 6:**
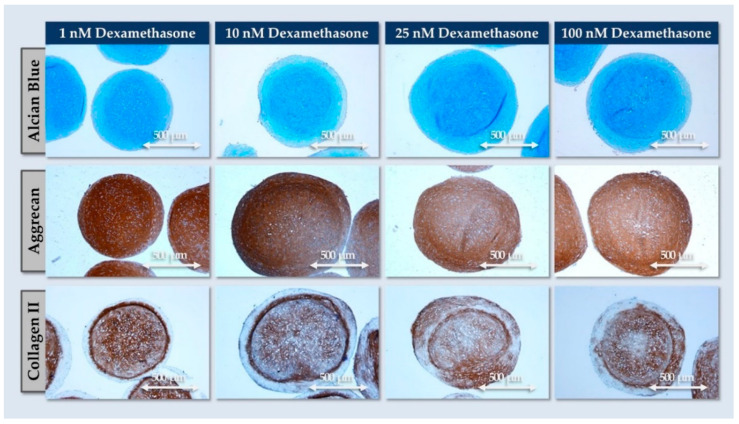
Results of FE002-Disc progenitor cell spheroid functional optimization assays, with comparative endpoint histological assessment of various dexamethasone concentrations in the chondrogenic induction medium. Scale bars = 500 µm.

The results presented in Figure 5 confirmed that the dexamethasone concentration in the chondrogenic induction medium impacted COMP expression and GAG contents, but these quantitative analyses did not allow to evaluate the quality (e.g., homogeneity) of matrix deposition in the considered spheroids. Therefore, histology was performed to observe ECM deposition. Therein, all dexamethasone concentrations resulted in strong endpoint AB and ACAN staining throughout the spheroids (Figure 6).

Of note, collagen II was also significantly expressed, but not throughout the spheroids (i.e., formation of an inner matrix-rich core surrounded by a dense cellular outer layer; Figure 6). Importantly, COL2 deposition was affected by the dexamethasone concentration, where a better homogeneity of staining was recorded with reduced concentrations (i.e., 1–10 nM; Figure 6). Based on the compilation of the obtained functional data for FE002-Disc cell spheroid formulation, a relatively low dose of 10 nM dexamethasone was retained in the chondrogenic induction medium. This choice was based on quantitative aspects of ECM component deposition in the spheroids, as well as qualitative aspects of ECM distribution.

Finally, it is noted that despite the absence of systematic verification of cellular viability throughout the spheroid culture period (e.g., ATP-based assays), the presence of metabolically active and functional cells throughout the culture phase was indirectly confirmed. Namely, the consistent increase in the size of the spheroids over time was due to the synthesis of ECM by viable IVD cells, and strong deposition of ECM components throughout the spheroid body over time was recorded (Figure 5 and Figure 6). Such facts presupposed the presence of viable cells in the spheroids, which are the only effectors capable of producing and structuring ECM in the 3D spheroids. Notwithstanding, validation of endpoint cellular viability maintenance in the spheroids is necessary for a finished product, including following transport and storage, yet the scope of the present work was limited to preclinical safety and function qualification, with functional optimization focusing on the cellular active substance and the in vitro process. Full validation campaigns, including ATP-based assays for release and stability testing, may be the focus of the next step of experimental work, as FE002-Disc cells have been preliminarily qualified herein.

### 3.6. FE002-Disc Progenitor Cell Spheroid Stability in Cryopreservation and Lyopreservation

**Figure 7:**
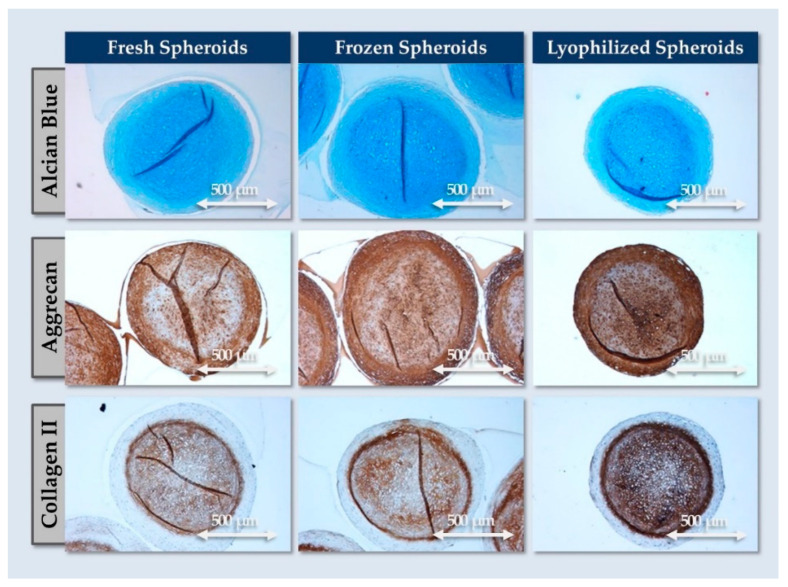
Results of FE002-Disc progenitor cell spheroid behavior following cryopreservation and lyopreservation, with histological assessment of critical structural attributes and ECM quality. Cell spheroids were manufactured using 10 nM dexamethasone. Cell spheroids manufactured using 100 nM dexamethasone are represented in Figure S8. Scale bars = 500 µm. ECM, extracellular matrix.

In order to preliminarily assess the potential of obtaining off-the-freezer or off-the-shelf FE002-Disc spheroids, two stabilization protocols were evaluated. The results indicated that all samples retained macroscopic structural integrity after freezing and lyophilization processing, respectively. Furthermore, histology readouts showed that no loss of ECM was sustained during freezing or lyophilization. Specifically, the spheroids all stained positive for AB, COL2, and ACAN (Figure 7).

Overall, the functional results obtained with frozen or lyophilized spheroids were interpreted positively from a compositional standpoint (i.e., no deterioration of structure or ECM; Figure 7 and Figure S8). Namely, no adverse effects were recorded in the stabilized groups as regards the quality of the components compared to the freshly harvested spheroids. Such protocols thus bare potential for the formulation of stable and consistent sources of exogeneous ECM components or for the further development of stabilized chondrogenic microtissues. In detail, the conservative nature of the retained pilot stabilization techniques enables us to consider further processing options (e.g., supercritical CO_2_ decellularization, terminal sterilization) for the investigated biological materials.

## 4. Discussion

### 4.1. Cell Therapies as Promising Contenders for IVD Pathology Management

The promise of cell-based therapies in IVD degeneration is linked to their multifaceted MoA, which has the potential to address the complexity of disc degeneration [64,65]. Importantly, current conservative treatments help in treating LBP symptoms but do not satisfactorily restore the disc environment, leading to further disc degeneration in the long term. Thus, the goal of cell-based therapies consists in regenerating disc structure or function and reducing LBP in patients. In the available clinical reports, IVD cell therapies were shown to positively impact pain symptoms and reduce disability [66,67,68]. However, mixed outcomes were reported as regards regenerative aspects (e.g., restoration of IVD height, hydration balance) of the interventions [3,8]. Nonetheless, chondrocytes and IVD cells appear to yield better outcomes for regeneration than alternative biologicals, suggesting that such cell types are better suited for implantation in adverse microenvironments [52,53,54,55].

Generally, several hurdles limit the practical identification and benchmarking of optimal cell sources for application in IVD therapies, as the specific MoA are not well-characterized. Selection criteria for therapeutic cellular contenders in investigational IVD treatments are notably based on current in vitro and in vivo data, as follows:

1. Safety attributes: absence of allergic or immunogenic reactions in the host, absence of tumorigenicity,
2. Chondrogenic/discogenic potential: synthesis and deposition of GAGs and collagen II in the implantation environment,
3. Cell sourcing: choice of discogenic cells (e.g., IVD cells, chondrocytes) with potent regeneration capacities and with environment modulatory attributes,
4. Cellular resilience following implantation: in situ resistance to harsh environmental constraints in vivo,
5. Supply chain considerations: allogeneic versus autologous starting biological materials,
6. Adaptability to GMP manufacturing processes: possibility to prepare and formulate therapeutic cells in a clinically deliverable product.

As specifically concerns the option to use allogeneic starting biological materials, more complex qualification steps are required compared to the use of autologous materials (e.g., adventitious agent screening). However, several quality and logistical benefits are procured by the use of allogeneic cell sources, such as reduced variability (i.e., no donor-to-donor influence) and the absence of an invasive harvesting surgery [34]. Based on the criteria and considerations presented hereabove, qualification testing was performed on the FE002-Disc cell source for preliminary demonstration of its applicability in IVD therapeutics.

### 4.2. Adequation of FE002-Disc Progenitor Cells with Cell Therapy Development Schemes

From a cell-based therapeutic product manufacturing standpoint, many elements must be closely considered and documented around the handling of biological materials. These comprise the methodology for qualification of the starting cell source, manufacturing process parameters, technical specifications for the process, and selection of critical materials [33]. Importantly, high scrutiny is allocated toward identifying and managing sources of variability during active substance or drug product manufacture [69]. Design approaches aim to minimize variability and to identify technical alternatives or variants in order to optimize finished product quality and performance [70]. In the respective domains of articular cartilage and IVD cell-based treatments, converging approaches of spheroid implantation in highly demanding host tissues appear most promising [51,52,53,54,71,72,73,74].

The experimental results gathered herein have notably confirmed the adequation of the FE002-Disc cell source with several requirements of cell therapy translational development. Overall, the investigated functional and safety attributes were assessed as being appropriate for potential formulation and use in IVD applications. Firstly, the sustainability of the cell source (i.e., based on the usable in vitro passage levels) was determined, as the therapeutic use of passage level 6–7 cells would enable to potentially treat thousands of patients. Secondly, the chondrogenic potential of the cells was validated, as effective chondrogenic differentiation (i.e., GAGs and collagen II production) was repeatedly observed (Figure 2 and Figure 6). Furthermore, it was confirmed that the cellular biological activity (i.e., chondrogenic potential) was reduced with in vitro cell aging (Figure 2).

Finally, in vitro cell safety validation was performed using telomerase activity assays, soft agarose assays, and confirmation of the limited lifespan of the primary FE002-Disc cell type. Specifically, the presented safety data were conjointly considered with the previously applied donor qualification panels (e.g., background anamnesis and adventitious agent screens) [33]. Overall, the gathered body of evidence was assessed to qualify the FE002-Disc cell source for further preclinical investigation (e.g., safety and efficacy studies in animal models).

### 4.3. Importance of Therapeutic Cell Resilience in Hypoxic and Inflammatory Environments

Generally, the specific constraints of the IVD implantation environment warrant the technical optimization of the cellular active substance and the finished product form. The degenerative IVD environment is notably hostile, with the absence of perfusion resulting in a hypoxic and glucose-deprived niche. Furthermore, pathological degeneration leads to acidification and inflammatory cytokine release, limiting the potential for exogenous cell survival and engraftment [9,10,75]. From a biological-based formulation viewpoint, integration of anti-inflammatory molecules (e.g., anti-TNF, anti-IL), growth factors, or cell preculture in hypoxia may be considered [76]. A specific difficulty in therapeutic protocol development lies in the sub-optimal modeling of degenerated human IVDs in animal models. Thus, efficiency parameters are often only assessed in human trials. With regard to the composition of the implantation environment, low-glucose and acidic in vitro models may be used to predict in situ cell survival [52,55,56]. Specifically, while the pH in healthy IVDs is neutral (i.e., 6.9–7.2), it drops to 6.0–6.2 in degenerated tissues due to anaerobic glycolysis. It was previously reported that NP stem cells survive better in acidic conditions than ASCs, as acidic conditions reduced cellular proliferation to a greater extent in ASCs [77].

From a more detailed mechanistic and functional viewpoint, nasal chondrocytes were exposed to adverse biochemical cues (i.e., inflammatory and hypoxic conditions) [78]. Therein, IL-1β treatment led to catabolic effects, with GAG loss, increased MMP-1 expression, and decreased COL2 [78]. Parallelly, while hypoxic culture conditions did not significantly impact GAGs, increased COL2 levels were evidenced [78]. In further studies, the authors sought an optimal chondrogenic cell source for implantation in IVDs (i.e., maintenance of viability and function in adverse environments) [52]. Therefore, MSCs, articular chondrocytes, and nasal chondrocytes were functionally benchmarked in hypoxic, acidic, inflammatory, and low-glucose conditions. Enhanced survival and ECM deposition of nasal chondrocytes was evidenced in hypoxic/low-glucose conditions, as compared to MSCs and articular chondrocytes [52]. Inflamed environments led to decreased GAG production in MSCs, while this parameter did not negatively impact articular and nasal chondrocytes. In contrast, medium acidification led to a dramatic reduction in GAG production and COL2 induction in all three cell types. Of note, MSCs were more affected than the chondrocytes, as pellets could not form in this environment. Overall, it was shown that MSCs were more vulnerable to harsh environments than articular and nasal chondrocytes, especially regarding chondrogenic properties [52].

Considering the highly specific physiological constraints of degenerative IVDs, various experimental assays were conducted to assess the in situ resilience of the FE002-Disc cells. Experimental designs were based on the references hereabove for articular and nasal chondrocytes. Specifically, it was shown that FE002-Disc cell monolayers were characterized by improved cell proliferation potential in hypoxia compared to normoxia, echoing previous reports on FE002-Ten primary progenitor tenocytes [79]. Furthermore, the present study confirmed that hypoxia (i.e., down to 2% O_2_) was a positive factor for cellular proliferation and differentiation (Figure 3 and Figure S6). Finally, it was confirmed that FE002-Disc cells were not negatively impacted following exposure to high TNF-α doses (i.e., in normoxia or hypoxia; Figure 4). Overall, this study confirmed that FE002-Disc cells were resilient toward key adverse drivers of IVD microenvironments. In order to further study the resilience of FE002-Disc cells, acidic and glucose-deprived culture conditions could be used to functionally investigate the formation and chondrogenic activity of the cell spheroids, which are expected to present enhanced resistance.

### 4.4. Clinical Advancements of Chondrogenic Cells for IVD Therapy

Due to important scientific interest and high market demand, various therapies and products for IVD treatment have been clinically and commercially brought forward. Notably, these have comprised both autologous and allogeneic therapeutic cell sources, where safety and efficacy data are available. Clinical application of the autologous Novocart DISC Plus product (i.e., expanded cells from herniated disc tissue) aimed to reduce degenerative sequelae following lumbar disk surgery or to prophylactically avoid adjacent disc degeneration [21,66]. The allogeneic MPC-06-ID treatment (i.e., mesenchymal precursor cells, Mesoblast) was reported to be safe and effective in a phase 3 trial with 24 months of follow-up [80]. Specifically, single injections of 6 × 10^6^ cells resulted in reductions in VAS scores and improved function (i.e., ODI and EQ-5D indexes), most probably through paracrine anti-inflammatory effects.

In another clinical trial on 182 patients, Vivex Biologics investigated the intradiscal delivery (i.e., 20 G spine needle) of allogeneic NP matrix, showing improved VASPI and ODI scores at 12 months (i.e., 54% pain improvement, 53% ODI improvement) [81]. An allogeneic discogenic cell-based approach (i.e., rebonuputemcel, DiscGenics) was tested in early or moderate degenerative IVD cases [68,72]. From a formulation viewpoint, it was shown that fresh and frozen injectable cell-based products could both be effectively used in vivo [67]. Importantly, dosing considerations indicated that lower doses of cells were therapeutically preferrable, possibly due to the adverse impacts of large numbers of apoptotic cells in situ [67]. For rebonuputemcel, phase 3 clinical results were reported in 2023, with improvements in low back pain, function, disc volume, and patient quality of life [82]. Optimal results were obtained with doses of 9 × 10^6^ cells/mL in hyaluronic acid and cryopreservation excipients, with clinical benefit maintenance after two years [82]. Finally, various alternative clinical-stage examples of cell-based treatments for IVD applications may be set forth, such as CordSTEM-DD (i.e., allogeneic umbilical cord-derived mesenchymal stem cells, CHABiotech), BRTX-100 (i.e., autologous stem cells, BioRestorative), or the RESPINE EU project (i.e., allogeneic BM-MSC) [83,84,85,86,87].

### 4.5. Cell Formulation Optimization for Injectable IVD Treatments

Based on the technical considerations presented hereabove and on the clinical requirements of IVD cases, adaptation of the therapeutic cell formulation attributes may be necessary. Specifically, manufacturing process variants may be used for a given cell source based on the target functional attributes of the implanted materials. The first approach (i.e., technically simpler) consists in the formulation of the expanded cells in suspension form (i.e., with liquid or gel vehicles). Therein, manufacturing processes are notably faster, easier to scale up, and do not require chemical induction. However, this approach is limited by the fact that the cells are dedifferentiated (i.e., no COL2 expression), most sensitive to the harsh implantation environment, and subject to potential cell leakage out of the treated IVD. A potential mitigation measure could consist in the injection of discogenic cells under an appropriate matrix or scaffold, similarly to existing clinical practice in knee articular cartilage chondrotherapy [21,56].

The second approach, which was experimentally retained herein, consists in the generation of chondrogenically matured three-dimensional constructs (i.e., cell spheroids). Specifically, in addition to the selection of a robust therapeutic cell source, such formulation means may be leveraged to further increase the resistance of the cellular payload to harsh implantation environments, as previously described [54,55]. Indeed, it is well known that cells in 2D are much more drug-sensitive than in 3D structures (e.g., spheroids, organoids) [88,89]. Thus, implanted cells are in all probability more protected in 3D structures than in single-cell suspensions. Therein, one of the simplest options is the preparation of cell spheroids with appropriate physical (e.g., size) and functional (e.g., ECM deposition) attributes [53]. In addition to providing physical protection, the 3D structure recapitulates the three-dimensional native cellular environment, which is required for chondrogenic differentiation and the production of GAGs and COL2, both of which are major constituents of the NP. Notably, cell spheroids have been investigated in tissue engineering for the treatment of various tissues (e.g., bone, cartilage, trachea, nerve conduit) [71]. Furthermore, various notable clinical trials using cell spheroids are underway in multiple therapeutic indications (Table 2).

A notable example of chondrogenic spheroids for spine treatment is the “nose to spine” approach, leveraging the aforementioned nasal chondrocytes [52,53]. Therein, cell spheroids (i.e., 12.5 × 10^3^ cells/spheroid) were cultured/induced in hypoxia (i.e., 5% O_2_) for 3–7 days and were formulated for spinal injection (i.e., <600 μm spheroids for use in 22G needles). Specifically, it was shown that these spheroids supported the administration process and were able to fuse with NP spheroids [53]. Furthermore, spheroid formation appears to increase the adhesion properties of the product, thereby reducing cell leakage risks once the spheroids are injected [54]. Interestingly, Kasamkattil et al. directly compared the effects of nasal chondrocyte cell suspensions or spheroid-based formulations in an in vitro degenerative disc disease microtissue NP model [55]. The 3D formulation was shown to increase GAG contents in NP spheroids compared to the cell suspension [55]. Furthermore, a reduction in IL-8 secreted by the NP model was stronger with nasal chondrocyte spheroids in early timepoints [55].

Alternative notable examples of autologous chondrogenic spheroids may be cited, notably for articular chondral defect management [49,51,90]. Therein, products and candidates such as Spherox^®^ or Cartibeads^®^ are prepared in vitro and clinically delivered on the lesion [51,90]. Based on the available evidence on chondrogenic cell spheroids for various therapeutic applications, this formulation approach was retained for the investigated FE002-Disc cells. Of note, a relatively low cell amount/spheroid (i.e., 3 × 10^4^ cells) was retained based on administration-related spheroid size requirements. It was confirmed herein that the progenitor cells of interest could be effectively formulated as spheroids, with excellent GAG deposition and COL2 induction (Figure 6). However, the need to further understand and optimize the production process (e.g., induction medium composition, hypoxia degree) was clearly evidenced herein. Indeed, the O_2_ level set at 2% significantly influenced the development of spheroid size, while dexamethasone levels influenced ECM composition (Figure 5, Figure 6 and Figure S6). Thus, additional work is warranted to notably fully understand and characterize the different parameters (i.e., key and critical parameters) for the production of this formulation type. In particular, process transposition towards clinical grade conditions (e.g., qualified consumables and ancillary materials) will require thorough testing and validation.

### 4.6. Study Limitations and Future Perspectives

The main identified limitations of the present study were related to the number of retained experimental readouts. In particular, the endpoint cellular viability and cell-based functionality (e.g., spheroid fusion assays) attributes were set out of the scope of this study. In detail, for strict consideration of the presented spheroids as a finished product prototype, appropriate formulation, stability, and administration studies would need to be performed. Notwithstanding, the applied functional readouts enabled to indirectly confirm the presence of viable and functional cells in the produced spheroids, which were able to synthesize and deposit ECM components. Furthermore, while the presented datasets did not disqualify the FE002-Disc cell source for quality or safety reasons, further preclinical assessment phases are required (e.g., in vivo work) from a regulatory standpoint [91,92,93,94]. Additionally, the present study focused mainly on one therapeutic primary cell type, whereas further validation studies may include additional comparator progenitor cell types and patient-derived adult IVD cell types for appropriate functional benchmarking.

Future research perspectives for this study comprise in-depth functional assessments of the cryopreserved and lyophilized versions of the presented spheroids. In detail, preclinical assays could be designed in order to study the influence of stabilization processes on the function of the spheroids (e.g., ability to fuse to target microtissues). Furthermore, based on the prior application of multiple sources of chondrogenic cells (e.g., nasal chondrocytes or articular chondrocytes) for spheroid-based chondrotherapies and/or IVD therapies, alternative progenitor chondrogenic cells (e.g., FE002-Cart.Art/FE002-Cart cell sources) could be investigated for similar purposes.

## 5. Conclusions

The present study aimed to preclinically qualify FE002-Disc cells and cell spheroids for potential IVD therapeutic applications based on safety and function-oriented assays. The results showed the sustainability of the considered clinical-grade cell source, which was functionally qualified up to passage level 7. Furthermore, this study demonstrated that FE002-Disc cells were well-adapted to growth in adverse environments, as modeled by hypoxia and inflammatory setups. Namely, the in vitro resilience of the considered chondrogenic cells was evidenced, and the critical importance of appropriately establishing the process technical specifications (e.g., incubation atmosphere, dexamethasone concentration) was set forth. Generally, this study enabled to enhance the quality of the obtained FE002-Disc spheroids from a functional viewpoint, with positive pilot indications about finished product storage possibilities (i.e., in frozen or in dry form). Overall, both the applicability of the FE002-Disc cell source and the adequation of the cell spheroid manufacturing process with therapeutic product preclinical development requirements were documented herein. Such elements were considered positively for the further investigation of the therapeutic potential of primary progenitor cell sources in musculoskeletal regenerative medicine.

## Figures and Tables

**Table 1:** Physical characterization results of FE002-Disc spheroids produced with 10 nM and 25 nM dexamethasone, as presented in Figure 5.

Parameter	Circularity		Feret’s Diameter (mm)		Roundness	
Dexamethasone Concentration (nM)	10 nM	100 nM	10 nM	100 nM	10 nM	100 nM
D7 Timepoint	0.47 ± 0.12	0.56 ± 0.10	0.98 ± 0.05	0.97 ± 0.02	0.95 ± 0.02	0.96 ± 0.01
D14 Timepoint	0.42 ± 0.16	0.46 ± 0.08	1.09 ± 0.02	1.08 ± 0.05	0.96 ± 0.02	0.97 ± 0.02
D21 Timepoint	0.51 ± 0.08	0.54 ± 0.08	1.19 ± 0.02	1.24 ± 0.10	0.96 ± 0.01	0.88 ± 0.05

**Table 2:** Selected clinical trials dealing with cell spheroids. ASC, adipose stem cells; IPS, induced pluripotent stem cells; MSC, mesenchymal stem cells.

Clinical Trial ^1^	Spheroids/Cell Type	Therapeutic Indications
NCT04262167	Autologous lung stem cells	Idiopathic pulmonary fibrosis
NCT04945018	Allogenic IPS cardiomyocytes	Heart failure
NCT05011474	Autologous matrillin-3 pre-treated ASCs	Disc degeneration
NCT05712148	MSCs	Retinitis pigmentosa
NCT04818203	Autologous dermal fibroblasts	Periorbital wrinkles

^1^ Clinical trial reference numbers are available on https://www.clinicaltrials.gov/, accessed on 2 September 2024.

## Data Availability

The data presented in this study are available within the article files.

## Supplementary Materials

The following supporting information can be downloaded at: https://www.mdpi.com/article/10.3390/pharmaceutics16101274/s1. Supplementary Methods: Antibody reference numbers; Figure S1: Comparative cell proliferation results; Figure S2: Complementary data for HIF-1 Western blotting; Figure S3: Cellular lifespan characterization results; Figure S4: Telomerase activity quantification results; Figure S5: Soft agarose transformation assay results; Figure S6: Cell spheroid manufacturing protocol optimization results; Figure S7: Complementary data for COMP Western blotting; Figure S8: Data on cell spheroid behavior in cryopreservation and lyopreservation studies; Table S1: Results of flow cytometry characterization assays; Table S2: Results of proteomic characterization assays.

## Abbreviations

AB	Alcian blue
ACAN	aggrecan
AF	annulus fibrosus
ASC	adipose-derived stem cells
ATMP	advanced therapy medicinal product
BCA	bicinchoninic acid
BMSC	bone marrow stromal cells
CD	cluster of differentiation
CH	Helvetic Confederation
CHUV	Centre hospitalier universitaire vaudois
CMtc	complete growth medium
COL	collagen
COMP	cartilage oligomeric matrix protein
CT	cycle threshold
DDD	degenerative disc disease
DMEM	Dulbecco’s modified Eagle medium
DMMB	dimethylmethylene blue assay
DMSO	dimethyl sulfoxide
DNA	deoxyribonucleic acid
EC	European Commission
ECL	electrochemiluminescence
ECM	extracellular matrix
FACS	fluorescence activated cell sorting
FBS	fetal bovine serum
FDA	US Food and Drug Administration
FGF	fibroblast growth factor
FITC	fluorescein isothiocyanate
GAG	glycosaminoglycan
GMP	good manufacturing practices
h	hour
HA	hyaluronic acid
HGF	hepatocyte growth factor
HIF-1	hypoxia-inducible factor
HLA	human leukocyte antigen
HMGB1	high mobility group protein B1
HPL	human platelet lysate
HRP	horseradish peroxidase
IBMX	3-isobutyl-1-methylxanthine
IL	interleukin
IPS	induced pluripotent stem cells
ITS	insulin, transferrin, selenous acid
IVD	intervertebral disc
IVDD	intervertebral disc disease
LBP	low back pain
M-CSF	macrophage colony-stimulating factor
min	minute
MMPs	matrix metalloproteinases
MoA	mechanism of action
MSC	mesenchymal stem cells
NA	non-applicable
NP	nucleus pulposus
NSAIDs	non-steroidal anti-inflammatory drugs
ODI	Oswestry disability index
PBS	phosphate-buffered saline
PDT	population doubling time
PDV	population doubling value
PE	phycoerythrin
PMSF	phenylmethylsulfonyl fluoride
PRP	platelet rich plasma
qPCR	quantitative polymerase chain reaction
TGF	transforming growth factor
TIMPs	tissue inhibitor of metalloproteinases
TNF	tumor necrosis factor
sEGFR	soluble epidermal growth factor receptor
sTNFRI	soluble tumor necrosis factor receptor I
UK	United Kingdom
USA	United States of America
USD	US dollar
VAS	visual analog scale
VASPI	visual analog scale pain intensity
VitCp	vitamin C 2-phosphate sesquimagnesium salt hydrate

## References

[1] Mohd Isa I.L., Teoh S.L., Mohd Nor N.H., Mokhtar S.A. (2022). Discogenic low back pain: Anatomy, pathophysiology and treatments of intervertebral disc degeneration. Int. J. Mol. Sci..

[2] Kim J.H., Ham C.H., Kwon W.K. (2022). Current knowledge and future therapeutic prospects in symptomatic intervertebral disc degeneration. Yonsei Med. J..

[3] Soufi K.H., Castillo J.A., Rogdriguez F.Y., DeMesa C.J., Ebinu J.O. (2023). Potential role for stem cell regenerative therapy as a treatment for degenerative disc disease and low back pain: A systematic review. Int. J. Mol. Sci..

[4] Meucci R.D., Fassa A.G., Faria N.M. (2015). Prevalence of chronic low back pain: Systematic review. Revista Saude Publ..

[5] Vos T., Flaxman A.D., Naghavi M., Lozano R., Michaud C., Ezzati M., Shibuya K., Salomon J.A., Abdalla S., Aboyans V. (2012). Years lived with disability (YLDs) for 1160 sequelae of 289 diseases and injuries 1990–2010: A systematic analysis for the Global Burden of Disease Study 2010. Lancet.

[6] Knezevic N.N., Candido K.D., Vlaeyen J.W.S., Zundert J.V., Cohen S.P. (2021). Low back pain. Lancet.

[7] Dagenais S., Caro J., Haldeman S. (2008). A systematic review of low back pain cost of illness studies in the United States and internationally. Spine J..

[8] Binch A.L.A., Fitzgerald J.C., Growney E.A., Barry F. (2021). Cell-based strategies for IVD repair: Clinical progress and translational obstacles. Nat. Rev. Rheumatol..

[9] Freemont A.J. (2009). The cellular pathobiology of the degenerate intervertebral disc and discogenic back pain. Rheumatology.

[10] Smith L.J., Silverman L., Sakai D., Le Maitre C.L., Mauck R.L., Malhotra N.R., Lotz J.C., Buckley C.T. (2018). Advancing cell therapies for intervertebral disc regeneration from the lab to the clinic: Recommendations of the ORS spine section. JOR Spine.

[11] Sharifi S., Bulstra S.K., Grijpma D.W., Kuijer R. (2015). Treatment of the degenerated intervertebral disc; closure, repair and regeneration of the annulus fibrosus. J. Tissue Eng. Regen. Med..

[12] Vo N.V., Hartman R.A., Yurube T., Jacobs L.J., Sowa G.A., Kang J.D. (2013). Expression and regulation of metalloproteinases and their inhibitors in intervertebral disc aging and degeneration. Spine J..

[13] Nicol V., Verdaguer C., Daste C., Bisseriex H., Lapeyre É., Lefèvre-Colau M.M., Rannou F., Rören A., Facione J., Nguyen C. (2023). Chronic low back pain: A narrative review of recent international guidelines for diagnosis and conservative treatment. J. Clin. Med..

[14] Pennicooke B., Moriguchi Y., Hussain I., Bonssar L., Härtl R. (2016). Biological treatment approaches for degenerative disc disease: A review of clinical trials and future directions. Cureus.

[15] Fritzell P., Hägg O., Wessberg P., Nordwall A., Swedish Lumbar Spine Study Group (2001). 2001 Volvo Award Winner in Clinical Studies: Lumbar fusion versus nonsurgical treatment for chronic low back pain: A multicenter randomized controlled trial from the Swedish Lumbar Spine Study Group. Spine.

[16] Mowbray J., Shen B., Diwan A.D. (2021). Intradiscal therapeutics for degenerative disc disease. Handbook of Spine Technology.

[17] Clinical Trial N°NCT01124006 A Multicenter, Randomized, Double-Blind, Placebo Controlled, Clinical Trial to Evaluate the Safety, Tolerability and Preliminary Effectiveness of 2 Doses of Intradiscal rhGDF-5 (Single Administration) for the Treatment of Early Stage Lumbar Disc Degeneration. NCT01124006.

[18] Clinical Trial N°NCT03122119 Effectiveness of Ultrasound Guided Platelet Rich Plasma Injections in the Sacroiliac Joint. NCT03122119.

[19] Zhang J., Liu D., Gong Q., Chen J., Wan L. (2022). Intradiscal autologous platelet-rich plasma injection for discogenic low back pain: A clinical trial. BioMed Res. Int..

[20] Clinical Trial N°NCT03955315 Study to Evaluate the Safety and Preliminary Efficacy of IDCT, a Treatment for Symptomatic Lumbar Disc Degeneration. NCT03955315.

[21] Tschugg A., Michnacs F., Strowitzki M., Meisel H.J., Thomé C. (2016). A prospective multicenter phase I/II clinical trial to evaluate safety and efficacy of NOVOCART Disc plus autologous disc chondrocyte transplantation in the treatment of nucleotomized and degenerative lumbar disc to avoid secondary disease: Study protocol for a randomized controlled trial. Trials.

[22] Amirdelfan K., Bae H., McJunkin T., DePalma M., Kim K., Beckworth W.J., Ghiselli G., Bainbridge J.S., Dryer R., Deer T.R. (2021). Allogeneic mesenchymal precursor cells treatment for chronic low back pain associated with degenerative disc disease: A prospective randomized, placebo-controlled 36-month study of safety and efficacy. Spine J..

[23] Gjefsen E., Bråten L.C.H., Goll G.L., Wigemyr M., Bolstad N., Valberg M., Schistad E.I., Marchand G.H., Granviken F., Selmer K.K. (2020). The effect of infliximab in patients with chronic low back pain and Modic changes (the BackToBasic study): Study protocol of a randomized, double blind, placebo-controlled, multicenter trial. BMC Musculoskel. Dis..

[24] Han F., Tu Z., Zhu Z., Liu D., Meng Q., Yu Q., Wang Y., Chen J., Liu T., Han F. (2023). Targeting endogenous reactive oxygen species removal and regulating regenerative microenvironment at annulus fibrosus defects promote tissue repair. ACS Nano.

[25] Wei Z., Ye H., Li Y., Li X., Liu Y., Chen Y., Yu J., Wang J., Ye X. (2024). Mechanically tough, adhesive, self-healing hydrogel promotes annulus fibrosus repair via autologous cell recruitment and microenvironment regulation. Acta Biomater..

[26] Herger N., Bermudez-Lekerika P., Farshad M., Albers C.E., Distler O., Gantenbein B., Dudli S. (2022). Should degenerated intervertebral discs of patients with modic type 1 changes be treated with mesenchymal stem cells?. Int. J. Mol. Sci..

[27] Sakai D., Andersson G.B. (2015). Stem cell therapy for intervertebral disc regeneration: Obstacles and solutions. Nat. Rev. Rheumatol..

[28] Sakai D., Schol J. (2017). Cell therapy for intervertebral disc repair: Clinical perspective. J. Orthop. Transl..

[29] Meisel H.J., Siodla V., Ganey T., Minkus Y., Hutton W.C., Alasevic O.J. (2007). Clinical experience in cell-based therapeutics: Disc chondrocyte transplantation. A treatment for degenerated or damaged intervertebral disc. Biomol. Eng..

[30] Meisel H.J., Ganey T., Hutton W.C., Libera J., Minkus Y., Alasevic O. (2006). Clinical experience in cell-based therapeutics: Intervention and outcome. Eur. Spine J..

[31] Krut Z., Pelled G., Gazit D., Gazit Z. (2021). Stem cells and exosomes: New therapies for intervertebral disc degeneration. Cells.

[32] Li Z., Wu Y., Tan G., Xu Z., Xue H. (2022). Exosomes and exosomal miRNAs: A new therapy for intervertebral disc degeneration. Front. Pharmacol..

[33] Laurent A., Hirt-Burri N., Scaletta C., Michetti M., de Buys Roessingh A.S., Raffoul W., Applegate L.A. (2020). Holistic approach of Swiss fetal progenitor cell banking: Optimizing safe and sustainable substrates for regenerative medicine and biotechnology. Front. Bioeng. Biotechnol..

[34] Philippe V., Jeannerat A., Peneveyre C., Jaccoud S., Scaletta C., Hirt-Burri N., Abdel-Sayed P., Raffoul W., Darwiche S., Applegate L.A. (2023). Autologous and allogeneic cytotherapies for large knee (osteo)chondral defects: Manufacturing process benchmarking and parallel functional qualification. Pharmaceutics.

[35] Darwiche S., Scaletta C., Raffoul W., Pioletti D.P., Applegate L.A. (2012). Epiphyseal chondroprogenitors provide a stable cell source for cartilage cell therapy. Cell Med..

[36] Wu H., Shang Y., Yu J., Zeng X., Lin J., Tu M., Cheang L.H., Zhang J. (2018). Regenerative potential of human nucleus pulposus resident stem/progenitor cells declines with ageing and intervertebral disc degeneration. Int. J. Mol. Med..

[37] Mabotuwana N.S., Rech L., Lim J., Hardy S.A., Murtha L.A., Rainer P.P., Boyle A.J. (2022). Paracrine factors released by stem cells of mesenchymal origin and their effects in cardiovascular disease: A systematic review of pre-clinical studies. Stem Cell Rev. Rep..

[38] Hung G., Ashvetiya T., Leszczynska A., Yang W., Hwang C.W., Gerstenblith G., Barth A.S., Johnston P.V. (2022). Paracrine-mediated rejuvenation of aged mesenchymal stem cells is associated with downregulation of the autophagy-lysosomal pathway. Npj Aging.

[39] Jarrige M., Frank E., Herardot E., Martineau S., Darle A., Benabides M., Domingues S., Chose O., Habeler W., Lorant J. (2021). The future of regenerative medicine: Cell therapy using pluripotent stem cells and acellular therapies based on extracellular vesicles. Cells.

[40] Wang C., Yu X., Yan Y., Yang W., Zhang S., Xiang Y., Zhang J., Wang W. (2017). Tumor necrosis factor-α: A key contributor to intervertebral disc degeneration. Acta Biochim. Biophys. Sin..

[41] Croft A.S., Guerrero J., Oswald K.A.C., Häckel S., Albers C.E., Gantenbein B. (2021). Effect of different cryopreservation media on human nucleus pulposus cells’ viability and trilineage potential. JOR Spine.

[42] Evenbratt H., Andreasson L., Bicknell V., Brittberg M., Mobini R., Simonsson S. (2022). Insights into the present and future of cartilage regeneration and joint repair. Cell Regen..

[43] Feng G., Li L., Liu H., Song Y., Huang F., Tu C., Shen B., Gong Q., Li T., Liu L. (2013). Hypoxia differentially regulates human nucleus pulposus and annulus fibrosus cell extracellular matrix production in 3D scaffolds. Osteoarthr. Cart..

[44] Bitterli T., Schmid D., Ettinger L., Krupkova O., Bach F.C., Tryfonidou M.A., Meij B.P., Pozzi A., Steffen F., Wuertz-Kozak K. (2023). Targeted screening of inflammatory mediators in spontaneous degenerative disc disease in dogs reveals an upregulation of the tumor necrosis superfamily. JOR Spine.

[45] Fan C., Wang W., Yu Z., Wang J., Xu W., Ji Z., He W., Hua D., Wang W., Yao L. (2024). M1 macrophage-derived exosomes promote intervertebral disc degeneration by enhancing nucleus pulposus cell senescence through LCN2/NF-κB signaling axis. J. Nanobiotechnol..

[46] Pan H., Li H., Guo S., Wang C., Long L., Wang X., Shi H., Zhang K., Chen H., Li S. (2023). The mechanisms and functions of TNF-α in intervertebral disc degeneration. Exp. Gerontol..

[47] Díaz L., Zambrano E., Flores M.E., Contreras M., Crispín J.C., Alemán G., Bravo C., Armenta A., Valdés V.J., Tovar A. (2020). Ethical considerations in animal research: The principle of 3R’s. Rev. Investig. Clin..

[48] Jeannerat A., Meuli J., Peneveyre C., Jaccoud S., Chemali M., Thomas A., Liao Z., Abdel-Sayed P., Scaletta C., Hirt-Burri N. (2023). Bio-enhanced neoligaments graft bearing FE002 primary progenitor tenocytes: Allogeneic tissue engineering & surgical proofs-of-concept for hand ligament regenerative medicine. Pharmaceutics.

[49] Mumme M., Barbero A., Miot S., Wixmerten A., Feliciano S., Wolf F., Asnaghi A.M., Baumhoer D., Bieri O., Kretzcchmar M. (2016). Nasal chondrocyte-based engineered autologous cartilage tissue for repair of articular cartilage defects: An observational first-in-human trial. Lancet.

[50] Almqvist K.F., Dhollander A.A., Verdonk P.C., Forsyth R., Verdonk R., Verbruggen G. (2009). Treatment of cartilage defects in the knee using alginate beads containing human mature allogenic chondrocytes. Am. J. Sports Med..

[51] Kutaish H., Tscholl P.M., Cosset E., Bengtsson L., Braunersreuther V., Mor F.M., Laedermann J., Furfaro I., Stafylakis D., Hannouche D. (2023). Articular cartilage repair after implantation of hyaline cartilage beads engineered from adult dedifferentiated chondrocytes: Cartibeads preclinical efficacy study in a large animal model. Am. J. Sports Med..

[52] Gay M.H., Mehrkens A., Rittmann M., Haug M., Barbero A., Martin I., Schaeren S. (2019). Nose to back: Compatibility of nasal chondrocytes with environmental conditions mimicking a degenerated intervertebral disc. Eur. Cells Mater..

[53] Gryadunova A., Kasamkattil J., Gay M.H.P., Dasen B., Pelttari K., Mironov V., Martin I., Schären S., Barbero A., Krupkova O. (2021). Nose to spine: Spheroids generated by human nasal chondrocytes for scaffold-free nucleus pulposus augmentation. Acta Biomater..

[54] Kasamkattil J., Gryadunova A., Martin I., Barbero A., Schären S., Krupkova O., Mehrkens A. (2022). Spheroid-based tissue engineering strategies for regeneration of the intervertebral disc. Int. J. Mol. Sci..

[55] Kasamkattil J., Gryadunova A., Schmid R., Gay-Dujak M.H.P., Dasen B., Hilpert M., Pelttari K., Martin I., Schären S., Barbero A. (2023). Human 3D nucleus pulposus microtissue model to evaluate the potential of pre-conditioned nasal chondrocytes for the repair of degenerated intervertebral disc. Front. Bioeng. Biotechnol..

[56] McDonnell E.E., Wilson N., Barcellona M.N., Ní Néill T., Bagnall J., Brama P.A.J., Cunniffe G.M., Darwish S.L., Butler J.S., Buckley C.T. (2023). Preclinical to clinical translation for intervertebral disc repair: Effects of species-specific scale, metabolism, and matrix synthesis rates on cell-based regeneration. JOR Spine.

[57] Martinez I., Elvenes J., Olsen R., Bertheussen K., Johansen O. (2008). Redifferentiation of in vitro expanded adult articular chondrocytes by combining the hanging-drop cultivation method with hypoxic environment. Cell Transplant..

[58] Chijimatsu R., Kobayashi M., Ebina K., Iwahashi T., Okuno Y., Hirao M., Fukuhara A., Nakamura N., Yoshikawa H. (2018). Impact of dexamethasone concentration on cartilage tissue formation from human synovial derived stem cells in vitro. Cytotechnology.

[59] Demoor M., Ollitrault D., Gomez-Leduc T., Bouyoucef M., Hervieu M., Fabre H., Lafont J., Denoix J.M., Audigié F., Mallein-Gerin F. (2014). Cartilage tissue engineering: Molecular control of chondrocyte differentiation for proper cartilage matrix reconstruction. Biochim. Biophys. Acta.

[60] Branly T., Contentin R., Desancé M., Jacquel T., Bertoni L., Jacquet S., Mallein-Gerin F., Denoix J.M., Audigié F., Demoor M. (2018). Improvement of the chondrocyte-specific phenotype upon equine bone marrow mesenchymal stem cell differentiation: Influence of culture time, transforming growth factors and type i collagen siRNAs on the differentiation index. Int. J. Mol. Sci..

[61] Gómez-Leduc T., Desancé M., Hervieu M., Legendre F., Ollitrault D., de Vienne C., Herlicoviez M., Galéra P., Demoor M. (2017). Hypoxia is a critical parameter for chondrogenic differentiation of human umbilical cord blood mesenchymal stem cells in type I/III collagen sponges. Int. J. Mol. Sci..

[62] Enochson L., Brittberg M., Lindahl A. (2012). Optimization of a chondrogenic medium through the use of factorial design of experiments. BioRes. Open Access.

[63] Kipnes J., Carlberg A.L., Loredo G.A., Lawler J., Tuan R.S., Hall D.J. (2003). Effect of cartilage oligomeric matrix protein on mesenchymal chondrogenesis in vitro. Osteoarthr. Cart..

[64] Shi P., Chee A., Liu W., Chou P.H., Zhu J., An H.S. (2019). Therapeutic effects of cell therapy with neonatal human dermal fibroblasts and rabbit dermal fibroblasts on disc degeneration and inflammation. Spine J..

[65] Farhang N., Silverman L., Bowles R.D. (2020). Improving cell therapy survival and anabolism in harsh musculoskeletal disease environments. Tissue Eng. Part B Rev..

[66] Tschugg A., Diepers M., Simone S., Michnacs F., Quirbach S., Strowitzki M., Meisel H.J., Thomé C. (2017). A prospective randomized multicenter phase I/II clinical trial to evaluate safety and efficacy of NOVOCART disk plus autologous disk chondrocyte transplantation in the treatment of nucleotomized and degenerative lumbar disks to avoid secondary disease: Safety results of Phase I—A short report. Neurosurg. Rev..

[67] Hiraishi S., Schol J., Sakai D., Nukaga T., Erickson I., Silverman L., Foley K., Watanabe M. (2018). Discogenic cell transplantation directly from a cryopreserved state in an induced intervertebral disc degeneration canine model. JOR Spine.

[68] Silverman L.I., Heaton W., Farhang N., Saxon L.H., Dulatova G., Rodriguez-Granrose D., Flanagan F., Foley K.T. (2020). Perspectives on the treatment of lumbar disc degeneration: The value proposition for a cell-based therapy, immunomodulatory properties of discogenic cells and the associated clinical evaluation strategy. Front. Surg..

[69] Silverman L.I., Flanagan F., Rodriguez-Granrose D., Simpson K., Saxon L.H., Foley K.T. (2019). Identifying and managing sources of variability in cell therapy manufacturing and clinical trials. Regen. Eng. Transl. Med..

[70] Rodriguez-Granrose D., Zurawski J., Heaton W., Tandeski T., Dulatov G., Highsmith A.A., Conen M., Clark G., Jones A., Loftus H. (2021). Transition from static culture to stirred tank bioreactor for the allogeneic production of therapeutic discogenic cell spheres. Stem Cell Res. Ther..

[71] Chae S., Hong J., Hwangbo H., Kim G. (2021). The utility of biomedical scaffolds laden with spheroids in various tissue engineering applications. Theranostics.

[72] Silverman L.I., Dulatova G., Tandeski T., Erickson I.E., Lundell B., Toplon D., Wolff T., Howard A., Chintalacharuvu S., Foley K.T. (2020). In vitro and in vivo evaluation of discogenic cells, an investigational cell therapy for disc degeneration. Spine J..

[73] Yoon K.H., Yoo J.D., Choi C.H., Lee J., Lee J.Y., Kim S.G., Park J.Y. (2021). Costal chondrocyte-derived pellet-type autologous chondrocyte implantation versus microfracture for repair of articular cartilage defects: A prospective randomized trial. Cartilage.

[74] Yoon K.H., Park J.Y., Lee J.Y., Lee E., Lee J., Kim S.G. (2020). Costal chondrocyte-derived pellet-type autologous chondrocyte implantation for treatment of articular cartilage defect. Am. J. Sports Med..

[75] Teixeira G.Q., Riegger J., Gonçalves R.M., Risbud M.V. (2023). Editorial: Intervertebral disc degeneration and osteoarthritis: Mechanisms of disease and functional repair. Front. Bioeng. Biotechnol..

[76] Tonomura H., Nagae M., Takatori R., Ishibashi H., Itsuji T., Takahashi K. (2020). The potential role of hepatocyte growth factor in degenerative disorders of the synovial joint and spine. Int. J. Mol. Sci..

[77] Han B., Wang H.C., Li H., Tao Y.Q., Liang C.Z., Li F.C., Chen G., Chen Q.X. (2014). Nucleus pulposus mesenchymal stem cells in acidic conditions mimicking degenerative intervertebral discs give better performance than adipose tissue-derived mesenchymal stem cells. Cells Tissues Org..

[78] Scotti C., Osmokrovic A., Wolf F., Miot S., Peretti G.M., Barbero A., Martin I. (2012). Response of human engineered cartilage based on articular or nasal chondrocytes to interleukin-1β and low oxygen. Tissue Eng. Part A.

[79] Jeannerat A., Peneveyre C., Armand F., Chiappe D., Hamelin R., Scaletta C., Hirt-Burri N., de Buys Roessingh A., Raffoul W., Applegate L.A. (2021). Hypoxic incubation conditions for optimized manufacture of tenocyte-based active pharmaceutical ingredients of homologous standardized transplant products in tendon regenerative medicine. Cells.

[80] Clinical Trial N°NCT02412735 Placebo-Controlled Study to Evaluate Rexlemestrocel-L Alone or Combined with Hyaluronic Acid in Participants with Chronic Low Back Pain (MSB-DR003). NCT02412735.

[81] Beall D.P., Davis T., DePalma M.J., Amirdelfan K., Yoon E.S., Wilson G.L., Bishop R., Tally W.C., Gershon S.L., Lorio M.P. (2021). Viable disc tissue allograft supplementation; One- and two-level treatment of degenerated intervertebral discs in patients with chronic discogenic low back pain: One year results of the VAST randomized controlled trial. Pain Phys..

[82] Lorio M.P., Tate J.L., Myers T.J., Block J.E., Beall D.P. (2024). Perspective on intradiscal therapies for lumbar discogenic pain: State of the science, knowledge gaps, and imperatives for clinical adoption. J. Pain Res..

[83] Noriega D.C., Ardura F., Hernández-Ramajo R., Martín-Ferrero M.Á., Sánchez-Lite I., Toribio B., Alberca M., García V., Moraleda J.M., González-Vallinas M. (2021). Treatment of degenerative disc disease with allogeneic mesenchymal stem cells: Long-term follow-up results. Transplantation.

[84] Clinical Trial N°NCT04530071 Evaluation of Safety, Tolerability, and Efficacy of CordSTEM-DD in Patients with Chronic Low Back Pain. NCT04530071.

[85] Noriega D.C., Ardura F., Hernández-Ramajo R., Martín-Ferrero M.Á., Sánchez-Lite I., Toribio B., Alberca M., García V., Moraleda J.M., Sánchez A. (2017). Intervertebral disc repair by allogeneic mesenchymal bone marrow cells: A randomized controlled trial. Transplantation.

[86] Elabd C., Centeno C.J., Schultz J.R., Lutz G., Ichim T., Silva F.J. (2016). Intra-discal injection of autologous, hypoxic cultured bone marrow-derived mesenchymal stem cells in five patients with chronic lower back pain: A long-term safety and feasibility study. J. Transl. Med..

[87] Horizon 2020 Project. Regenerative Therapy of Intervertebral Disc: A Double Blind Phase 2b Trial of Intradiscal Injection of Mesenchymal Stromal Cells in Degenerative Disc Disease of the Lomber SPINE Unresponsive to Conventional Therapy. https://cordis.europa.eu/project/id/732163/results.

[88] Yan X., Zhou L., Wu Z., Wang X., Chen X., Yang F., Guo Y., Wu M., Chen Y., Li W. (2019). High throughput scaffold-based 3D micro-tumor array for efficient drug screening and chemosensitivity testing. Biomaterials.

[89] Flampouri E., Imar S., OConnell K., Singh B. (2019). Spheroid-3D and monolayer-2D intestinal electrochemical biosensor for toxicity/viability testing: Applications in drug screening, food safety, and environmental pollutant analysis. ACS Sens..

[90] Eschen C., Kaps C., Widuchowski W., Fickert S., Zinser W., Niemeyer P., Roël G. (2020). Clinical outcome is significantly better with spheroid-based autologous chondrocyte implantation manufactured with more stringent cell culture criteria. Osteoarthr. Cart. Open.

[91] Ikawa T., Yano K., Watanabe N., Masamune K., Yamato M. (2015). Non-clinical assessment design of autologous chondrocyte implantation products. Regen. Ther..

[92] Bukovac P.K., Hauser M., Lottaz D., Marti A., Schmitt I., Schochat T. (2023). The regulation of cell therapy and gene therapy products in Switzerland. Adv. Exp. Med. Biol..

[93] Kim J., Park J., Song S.Y., Kim E. (2022). Advanced therapy medicinal products for autologous chondrocytes and comparison of regulatory systems in target countries. Regen. Ther..

[94] Nordberg R.C., Otarola G.A., Wang D., Hu J.C., Athanasiou K.A. (2022). Navigating regulatory pathways for translation of biologic cartilage repair products. Sci. Transl. Med..

[95] World Medical Association (2013). Declaration of Helsinki: Ethical principles for medical research involving human subjects. JAMA.

