# Supplementary material for: Banked Primary Progenitor Cells for Allogeneic Intervertebral Disc (IVD) Therapy: Preclinical Qualification and Functional Optimization within a Cell Spheroid Formulation Process
Source: Pharmaceutics. 2024 Sep 29;16(10):1274. doi: 10.3390/pharmaceutics16101274 (PMC11510186; doi:10.3390/pharmaceutics16101274)
Supplement: Supplementary file 1 [file pharmaceutics-16-01274-s001.zip › pharmaceutics-3216135-supplementary.pdf]

Supplementary Materials:

Banked Primary Progenitor Cells for Allogeneic Intervertebral Disc (IVD) Therapy: Preclinical Qualification and Functional Optimization Within a Cell Spheroid Formulation Process

Annick Jeannerat, Cédric Peneveyre, Sandra Jaccoud, Virginie Philippe, Corinne Scaletta, Nathalie Hirt-Burri, Philippe Abdel-Sayed, Robin Martin, Lee Ann Applegate, Dominique P. Pioletti and Alexis Laurent

1. Supplementary Methods

1.1. Flow Cytometry Assays

Antibodies used for the flow cytometry analyses were all from BD Biosciences: CD90 (ref. 561970); CD73 (ref. 550257); CD105 (ref. 560839); CD19 (ref. 555413); CD34 (ref. 560941); CD14 (ref. 555397); CD45 (ref. 560976); CD44 (ref. 550989); CD26 (ref. 555437); CD166 (ref. 559263); MHC class I HLA-ABC (ref. 560964); MHC class II HLA-DPQR (ref. 555558); IgG1 isotype (ref. 555749); IgG1 isotype (ref. 554679); IgG2a isotype (ref. 555573).

2. Supplementary Figures

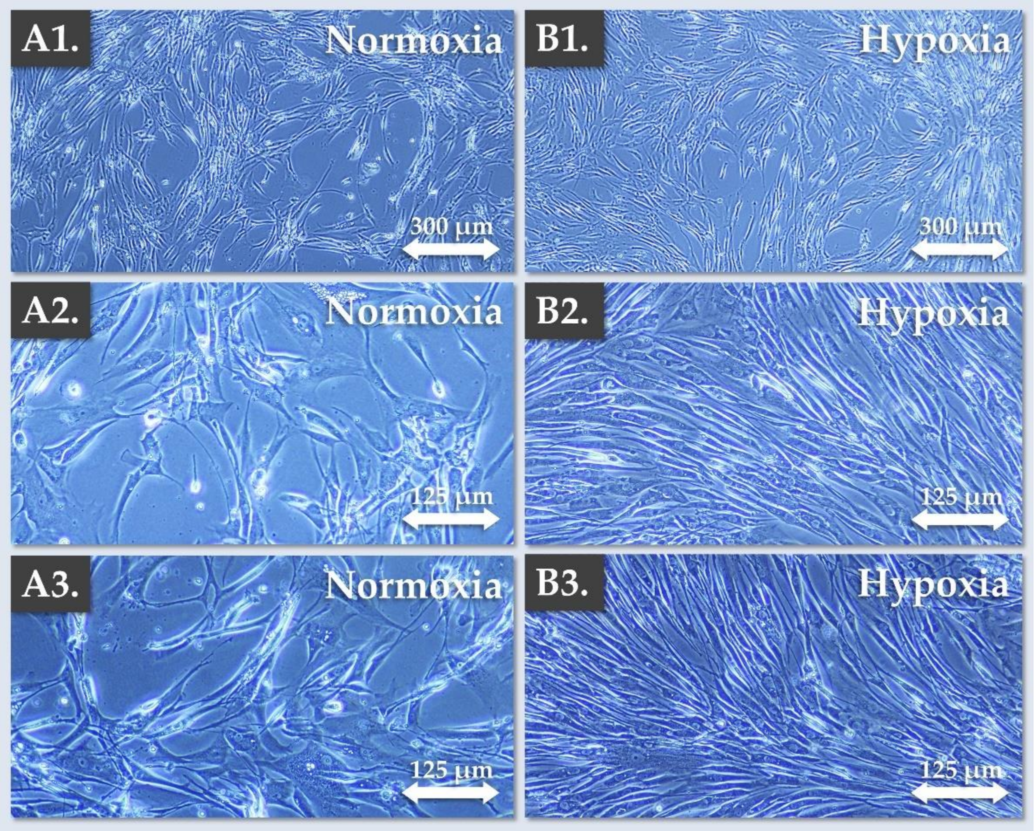

Figure S1. Results of comparative cell proliferation assays at passage level 7. (**A1**–**A3**) Random multi-field imaging of FE002-Disc primary progenitors proliferating in normoxic manufacturing conditions. (**B1**–**B3**) Random multi-field imaging of FE002-Disc primary progenitors proliferating in hypoxic manufacturing conditions. Scale bars = 125 µm or 300 µm.

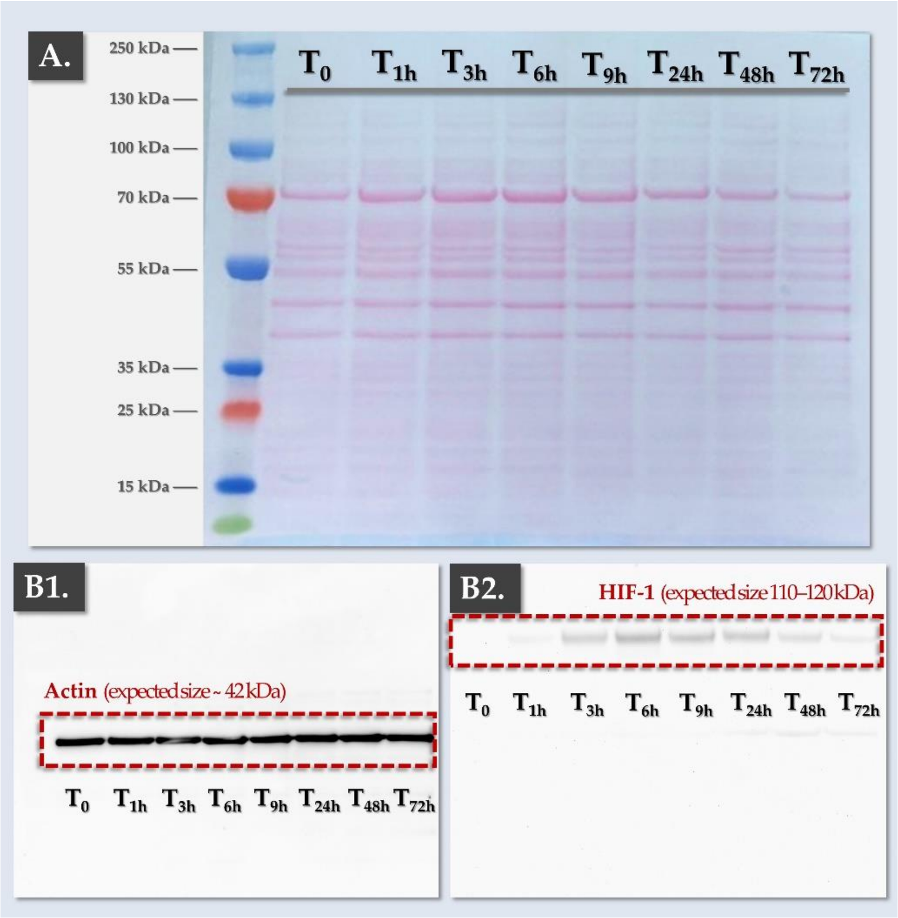

Figure S2. Whole gel imaging for the HIF detection assays reported in Figure 3. (**A**) Ponceau staining of the gel. (**B1**) ECL revelation of actin. (**B2**) ECL revelation of HIF-1. ECL, electrochemiluminescence; HIF, hypoxia-inducible factor; kDa, kilodalton.

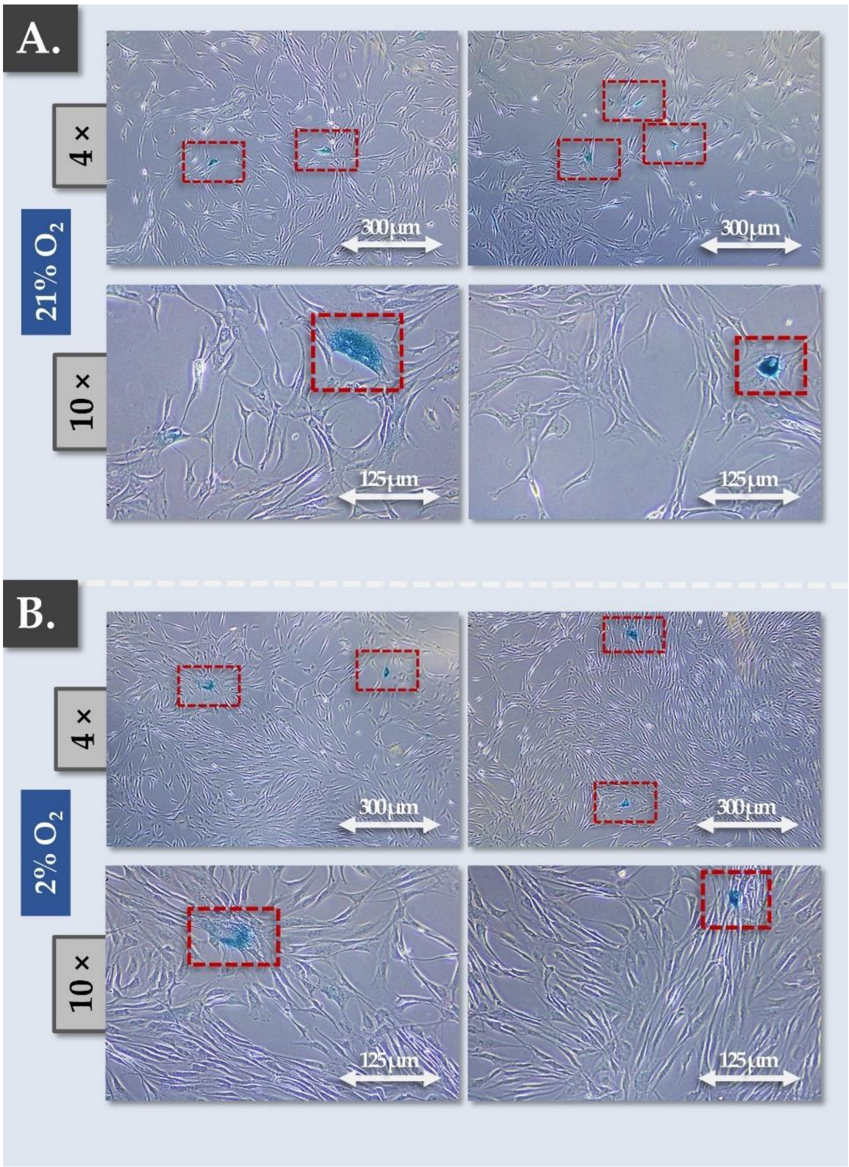

Figure S3. Results of in vitro cellular lifespan characterization assays for the cellular active substance. Cellular senescence was detected using β-galactosidase staining of proliferating FE002-Disc primary progenitor cells. Cells positive for β-gal staining (i.e., blue stain) were evidenced in red outlines. Scale bars = 125 µm or 300 µm. (**A**) Cells proliferating in normoxic manufacturing conditions, imaged under various optical enlargements. (**B**) Cells proliferating in hypoxic manufacturing conditions, imaged under various optical enlargements.

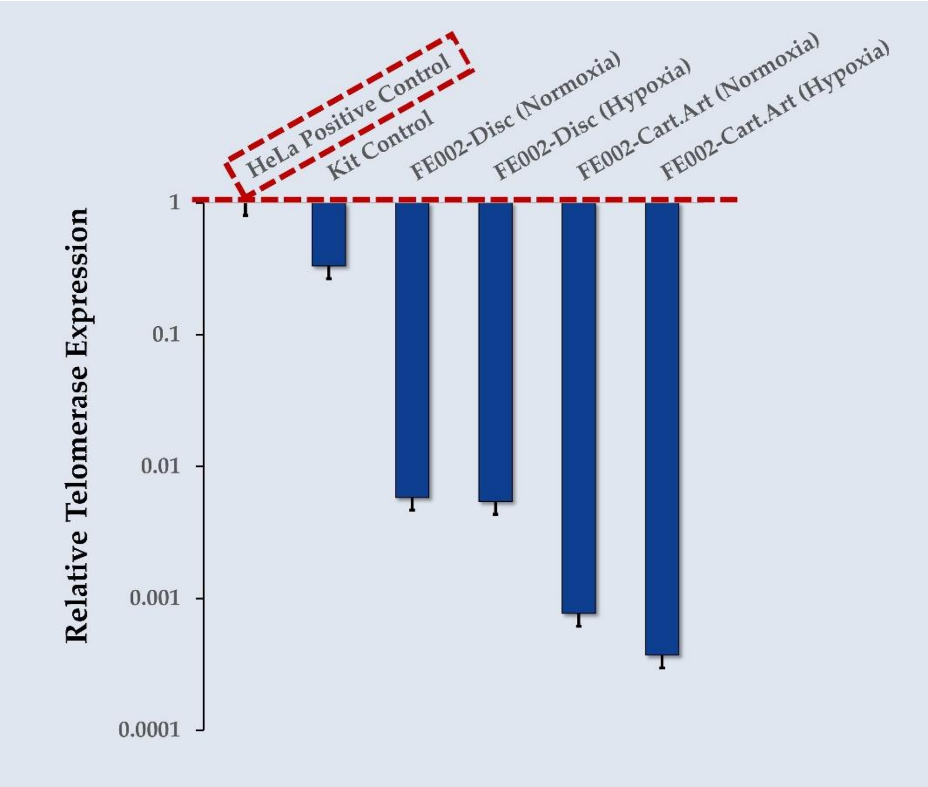

Figure S4. Results of telomerase expression quantification assays in target cells. Telomerase expression was quantified in FE002-Disc primary progenitors and in FE002-Cart.Art articular chondroprogenitors in both normoxic and hypoxic culture environments and was normalized to the positive control (i.e., HeLa cells). Both primary cell types presented a drastically reduced telomerase expression compared to HeLa cells.

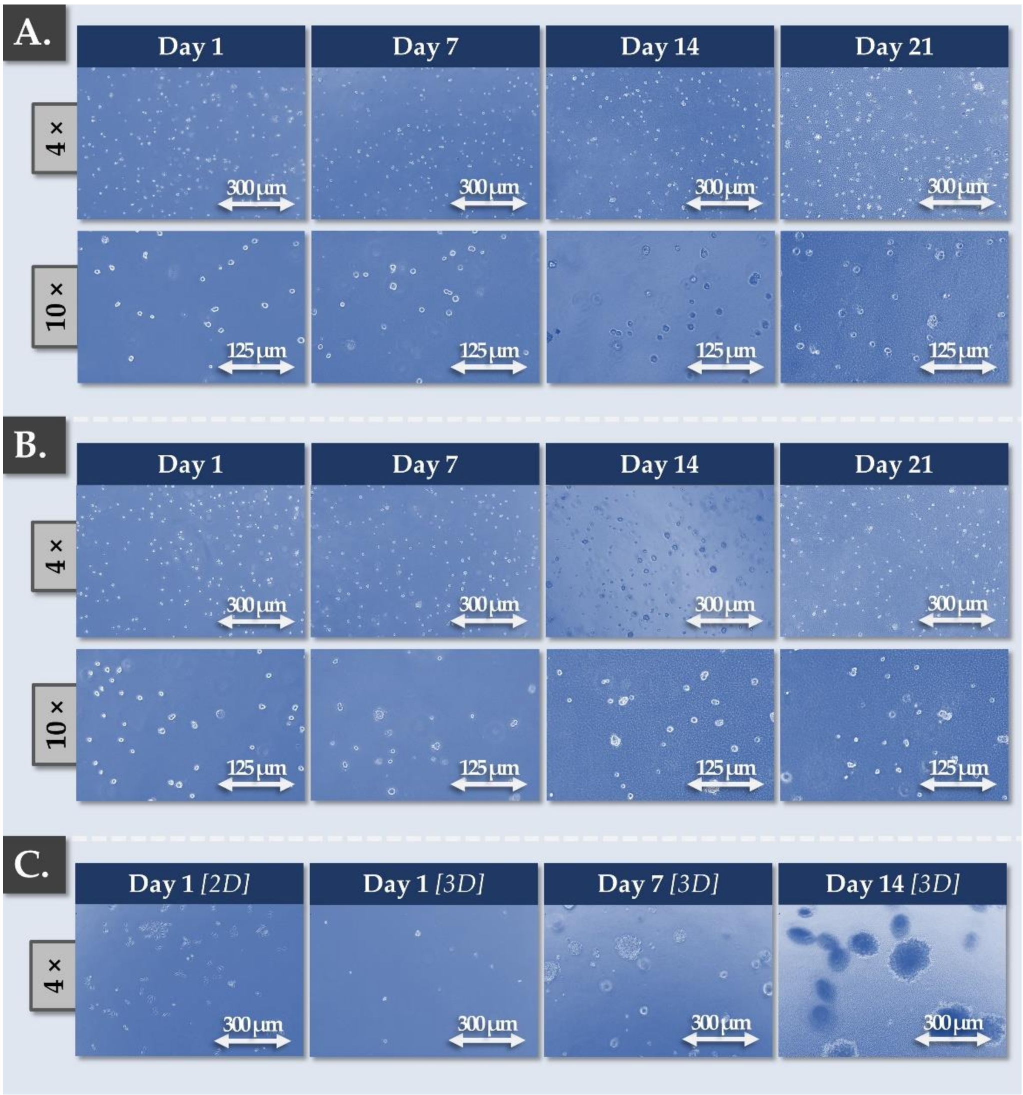

Figure S5. Results of soft agarose colony formation assays for the in vitro characterization of allogeneic cellular active substance safety attributes. Multiple timepoints of FE002-Disc primary progenitor cell non-adherent in vitro culture in soft agarose under various optical enlargements are presented. (**A**) Assay performed under normoxia conditions. (**B**) Assay performed under hypoxia conditions. No anchorage-independent FE002-Disc cellular proliferation was evidenced up to the final timepoint (i.e., day 21). (**C**) Multiple timepoints of HeLa cells (i.e., positive control) non-adherent in vitro culture in soft agarose under various optical enlargements. Cultures were stopped at the 14-day timepoint due to large cell colony size. Important anchorage-independent HeLa cellular proliferation was evidenced as early as the second timepoint (i.e., day 7). Scale bars = 125 μm or 300 μm.

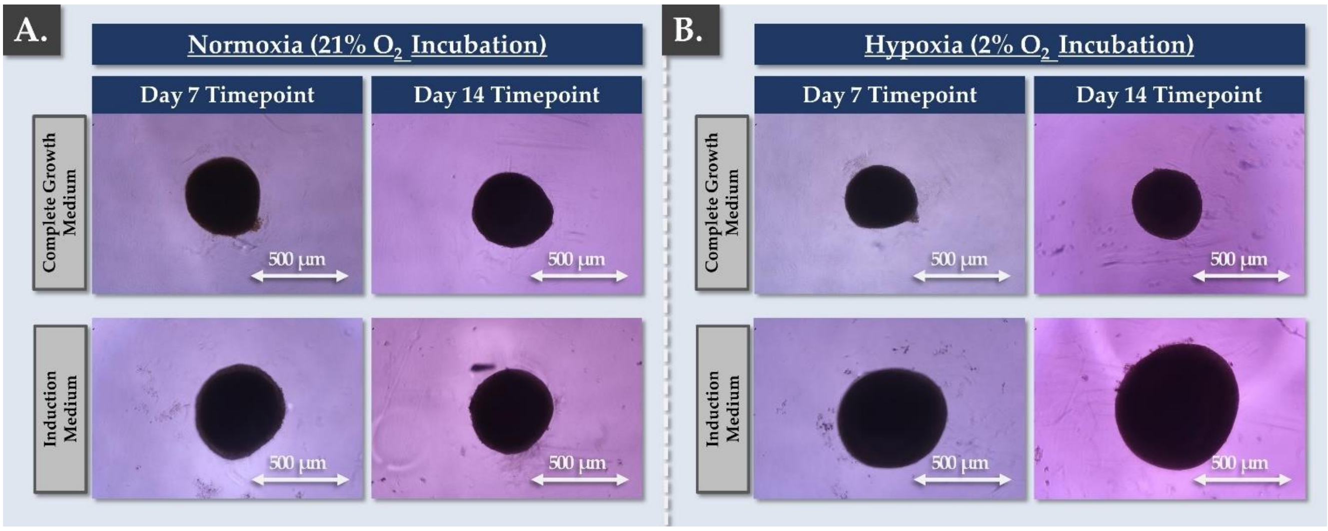

Figure S6. Results of cell spheroid preliminary manufacturing protocol optimization assays. (**A**) Spheroid macroscopic appearance after 7 days and 14 days of culture in normoxia in either complete growth medium or chondrogenic induction medium. No obvious change in spheroid size was visible. (**B**) Spheroid macroscopic appearance after 7 days and 14 days of culture in hypoxia in either complete growth medium or chondrogenic induction medium. A drastic increase in spheroid size over time was observed in hypoxia combined to chondrogenic induction medium. Scale bars = 500 µm.

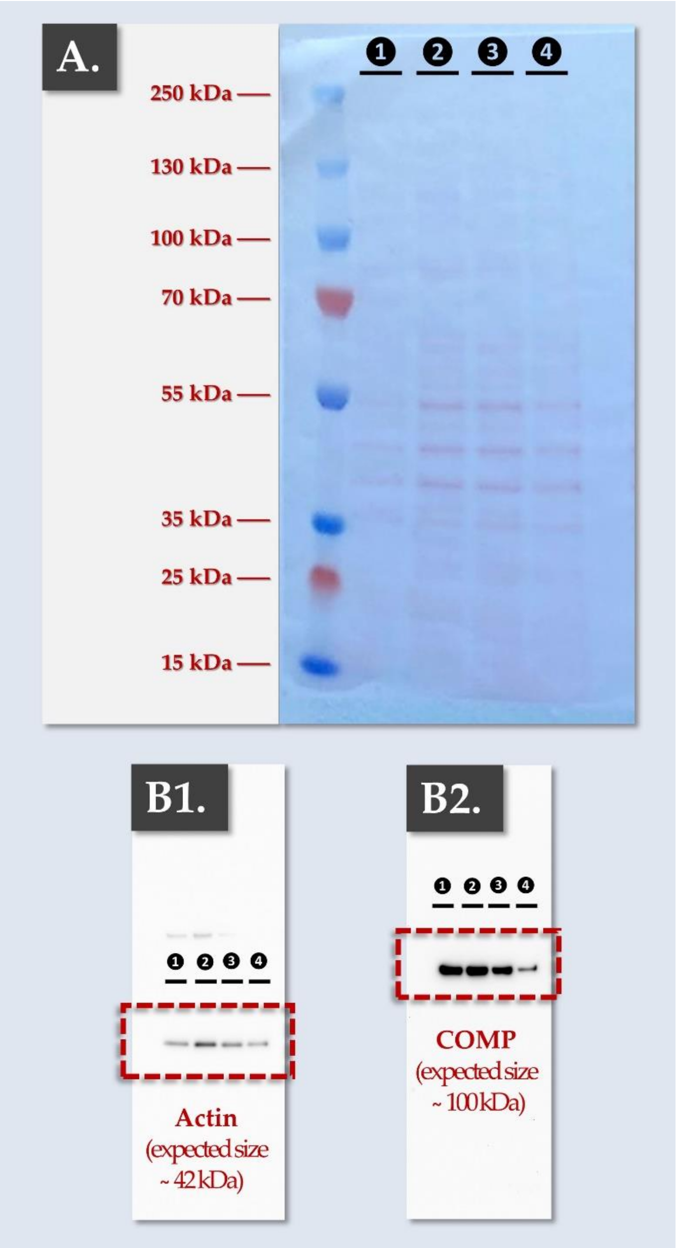

Figure S7. Whole gel imaging for the COMP assay reported in Figure 5. (**A**) Ponceau staining of the gel loaded with spheroid lysates obtained following induction with 100 nM, 25 nM, 10 nM, or 1 nM dexamethasone (i.e., columns 1 to 4). (**B1**) ECL revelation of actin. (**B2**) ECL revelation of COMP. COMP, cartilage oligomeric matrix protein; ECL, electrochemiluminescence; kDa, kilodalton.

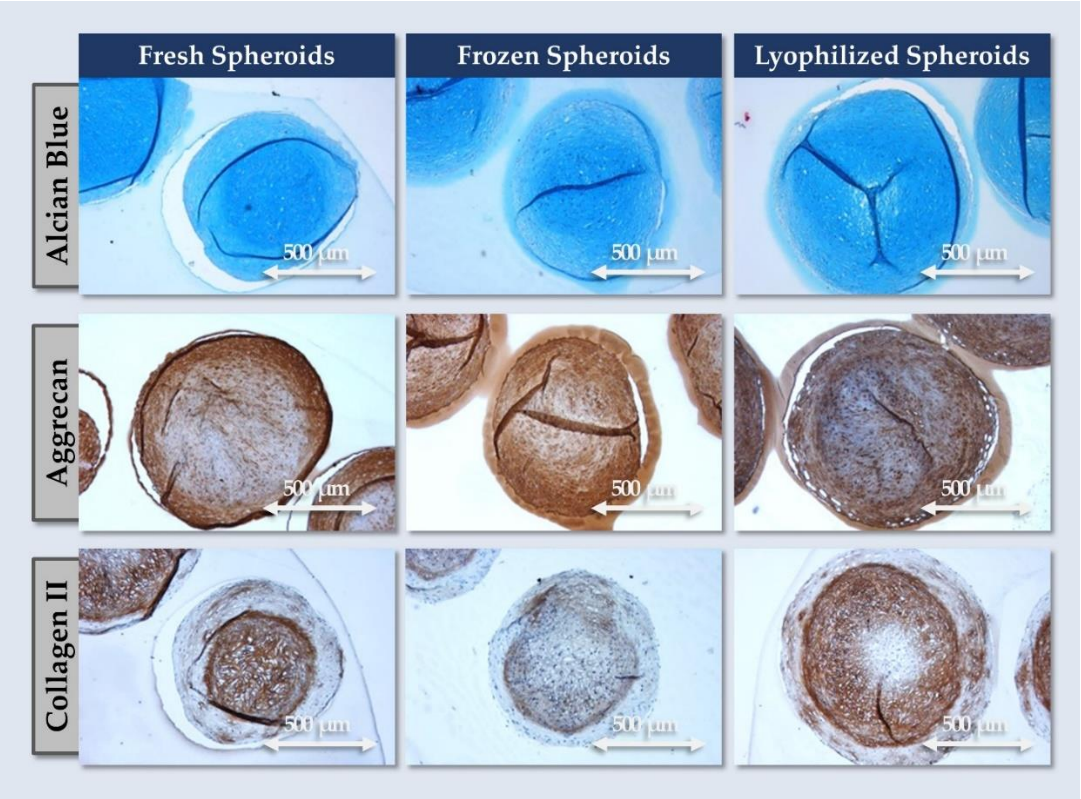

Figure S8. Results of cell spheroid behaviour in cryopreservation and lyopreservation. Cell spheroids were manufactured using 100 nM dexamethasone. Scale bars = 500 µm.

3. Supplementary Tables

Table S1. Results of flow cytometry characterization assays for FE002-Disc primary progenitor cells expanded in normoxia and in hypoxia, respectively. CD, cluster of differentiation; HLA, human leukocyte antigen.

Cell Surface Marker Type	Expression Result	
	Normoxia	Hypoxia
CD14	Negative	Negative
CD19	Negative	Negative
CD26	Positive	Positive
CD34	Negative	Negative
CD44	Positive	Positive
CD45	Negative	Negative
CD73	Positive	Positive
CD90	Positive	Positive
CD105	Positive	Positive
CD166	Positive	Positive
HLA-ABC	Positive	Positive
HLA-DPQR	Negative	Negative

Table S2. Results of proteomic characterization assays performed on FE002-Disc primary progenitor cell lysate, following monolayer expansion in normoxia and in hypoxia. The proteins with a normalized concentration > 0.5 ng/mg total protein are reported.

Protein Name (Abbreviated Name)	Normalized Relative Protein Quantity in the Cell Lysate Soluble Fraction (ng/mg) [1]	
	Normoxia	Hypoxia
Tissue inhibitor of metalloproteinases 2 (TIMP-2)	13.78	10.72
High-mobility group protein B1 (HMGB1)	12.76	20.31
Matrix metalloproteinase 2 (MMP-2)	8.28	4.45
Soluble epidermal growth factor receptor (sEGFR)	4.29	4.68
Soluble gp130 (sgp130)	3.92	5.32
Tissue inhibitor of metalloproteinases 1 (TIMP-1)	3.91	3.65
Hepatocyte growth factor (HGF)	1.55	1.48
Fibroblast growth factor 2 (FGF-2)	1.00	1.48
Soluble tumor necrosis factor receptor type I (sTNFRI)	0.80	1.15
Macrophage colony-stimulating factor (M-CSF)	0.61	0.52
Activin-binding protein (Follistatin)	0.60	0.95

[1] The relative protein quantity, as detected and normalized to the total protein content of the sample soluble fraction, was expressed in ng/mg.
